# Simultaneous real-time imaging of oxygen gradients and in vivo microbial community spatial organization in confined environments

**DOI:** 10.1093/ismeco/ycag146

**Published:** 2026-05-29

**Authors:** Giulia Ceriotti, Sergey M Borisov

**Affiliations:** Institute of Earth Surface Dynamics, Faculty of Geoscience and Environment, University of Lausanne, 1015 Lausanne, Switzerland; Institute of Analytical Chemistry and Food Chemistry, Faculty of Technical Chemistry, Chemical and Process Engineering and Biotechnology, Graz University of Technology, 8010 Graz, Austria

**Keywords:** (max 10): Oxygen gradients, microfluidics, luminescent sensors, microbial self-organization, porous media, fluorescent bacteria, microscale ecology, confined environments

## Abstract

Microorganisms in nature are often found in porous confined environments (soils, sediments, or plant and animal tissues), self-organized in heterogeneous communities. Such an organization, resulting from complex interactions and self-generated steep chemical gradients, controls the emergent microbial community’s ecological functions. The combination of microfluidics, fluorescent bacteria, and optical sensing can constitute a powerful tool to achieve new insights into these microbial dynamics. However, such a combination has remained challenging so far due to the limited compatibility of the current sensing approaches and fluorescent reporters. Here, we present a sensing microfluidic platform that enables simultaneous, real-time visualization of microscale oxygen gradients and multi-strain microbial community organization under flow. The approach combines transparent microfluidic devices, genetically encoded fluorescent bacteria, and the latest generation of near-infrared (NIR) luminescent oxygen sensors. We demonstrate that NIR oxygen sensing allows interference-free mapping of oxygen concentrations alongside GFP and mScarlet-I-labeled bacteria. Applying this platform to a heterogeneous porous geometry, we track the co-development of oxygen gradients and spatial organization in a two-strain *Pseudomonas* community under flowing conditions. The two strains exhibit distinct microscale spatial patterns consistent with known shape-dependent attachment and growth dynamics, while inducing sharp oxygen gradient formation. This work establishes a broadly accessible experimental framework for quantitatively linking microbial self-organization to chemical microenvironments in real time. This platform is cost-effective, customizable, and adaptable to sense different analytes, while being compatible with a range of spectrometry techniques. Therefore, this methodology opens new avenues for investigating microscale ecological processes in soils, sediments, and other confined habitats.

## Introduction

In nature, most microorganisms form structured communities and biofilms that enhance survival in hostile and competitive environments [1, 2]. Microbial communities, organized as heterogeneous mosaics of discrete colonies [3], are ubiquitous across ecosystems and often found in confined and inaccessible environments, including soils and sediments, deep or extreme habitats [4–6], and the tissues of plants and animals [7, 8]. Within these dense aggregates, multiple taxa and physiological states coexist and interact both with one another and with the surrounding physicochemical environment [1]. These interactions determine the emergent ecological functions of microbial communities and, ultimately, shape large-scale ecosystem processes [9, 10].

Responding to multiple coupled factors, including differences in metabolic and motility strategies, symbiotic, mutualistic, and predatory interactions, stochastic gene expression and mutation, and physical and chemical constraints [1, 11–15], microbial communities self-organize into heterogeneous structures that evolve dynamically across short temporal (hours) and spatial (10–100 μm) scales [14, 16–19]. In addition, microbial organization dynamically adjusts to self-induced physicochemical heterogeneities, such as increased local pressure and steep chemical gradients generated by multicellularity aggregation and nutrient consumption [1, 20–22].

Owing to the diversity of microbial life and the large number of interacting biotic and abiotic factors involved, the self-organization of microbial communities remains a complex and still incompletely understood phenomenon. Direct observation of natural community dynamics is challenging and potentially destructive, due to the inaccessibility of many microbial habitats and the limited spatial resolution of most imaging techniques [10, 20]. To overcome these limitations, microfluidic devices, that are transparent micromodels with customizable geometries, are attracting growing interest, allowing the real-time quantitative observation of microbial communities within confined environments using video microscopy [11, 20, 23].

In this context, microfluidic platforms become particularly powerful when combined with synthetic consortia and bioengineered, fluorescently tagged bacteria, which help discriminate optically between different phenotypes and strains [1, 23]. For example, experiments using fluorescent *Escherichia coli* mutants have elucidated the role of coupling between cell motility and heterogeneous pore-water velocities in porous media in determining cell attachment locations and colony formation [24]. Another example from medical microbiology used microfluidic devices mimicking epithelial tissues to reveal the critical role of extracellular polymeric substance (EPS) production in pathogen-driven mechanical disruption of host tissues as a potential mechanism of infection [7]. These studies exemplify how relatively simple experimental scenarios, high replicability, and fully controllable conditions elucidated the impact of biophysical factors on community dynamics. Although such systems cannot fully capture the complexity of natural communities, the combination of microfluidics and bioengineering is laying the foundation for a quantitative, physics-based understanding of microbial community organization at the microscale [1, 23, 25].

In parallel, sensing microfluidic devices, i.e., integrated with optical planar sensors or sensing nanoparticles [26], have been proposed to monitor chemical dynamics within microbial communities. Such tools have proven effective for visualizing the real-time formation of self-induced microscale oxygen (O_2_) gradients within and around microbial aggregates [21, 22, 27–32]. The combined use of O_2_-sensing microfluidics and bioengineered fluorescent bacteria holds great potential for elucidating how microbes reorganize spatially or activate anaerobic metabolic pathways in response to local O_2_ depletion. Pioneering studies have explored this direction. For example, using commercial planar (opaque) sensors, spatial segregation of aerobic and facultative anaerobic bacteria has been observed in response to self-generated carbon and O_2_ gradients [31]. Using sensing nanoparticles, Smriga et al. [21] demonstrated that spatially organized metabolic differentiation emerges within aggregates of facultative denitrifiers in response to self-induced O_2_ gradients.

However, the broader adoption of such approaches remains limited because the wide absorption and emission spectra of commonly used sensing dyes overlap with those of most fluorescent proteins employed in microbial bioengineering [33]. As a result, the presence of sensors often prevents visualization of fluorescently labeled cells while contributing to the photodegradation of fluorescent protein expressed by bacteria. Consequently, correlations between microbial organization and self-induced chemical gradients so far have relied on independent replicates of the experiments performed with and without sensors, followed by statistical comparisons.

**Figure 1:**
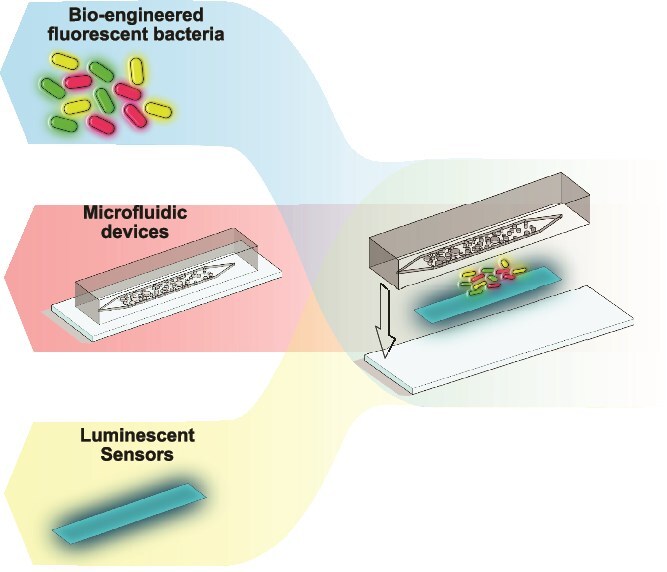
Graphical representation of the three tools (microfluidic devices, bioengineered fluorescent bacteria, and luminescent transparent sensor) that are combined in this work to provide full optical access to the ecology of microbial communities via fluorescence microscopy.

Here, we present a microfluidic approach that provides full optical access to real-time microscale O_2_ gradients generated by microbial aggregates and in vivo self-organization dynamics, simultaneously (Fig. 1). The system combines microfluidic devices with fully customizable geometries and the last generation of transparent O_2_ sensor based on a formulation that emits in the far-red to near-infrared (NIR) spectral range [34]. We demonstrate that this sensor enables the mapping of microscale O_2_ gradients induced by microbial activity without interfering with the fluorescent signal of commonly used fluorescent proteins. We apply this platform to a model system in which pore-water solute transport and shear stress within a heterogeneous porous geometry shape the spatial organization of a two-strain microbial community. We close by highlighting possible technical integrations that could further enhance the informational content of the proposed microfluidic platform.

The proposed setup, harnessing dyes that emit in the far-red range, retains the key properties of widely used luminescent sensors (eg, PtTFPP), namely photostability, reversible quenching, and versatility across applications, while enabling simultaneous detection of multiple bioengineered fluorescent strains. At the same time, it shares with all luminescent-based chemical imaging approaches some limitations, including the lack of standardized fabrication protocol and challenges in spatial resolution characterization. Despite these challenges yet to be overcome, altogether, our results highlight the potential of this approach for microbial ecology, emphasizing its replicability, cost-effectiveness, and adaptability to a wide range of ecological scenarios.

## Materials and methods

### Sensor production

**Figure 2:**
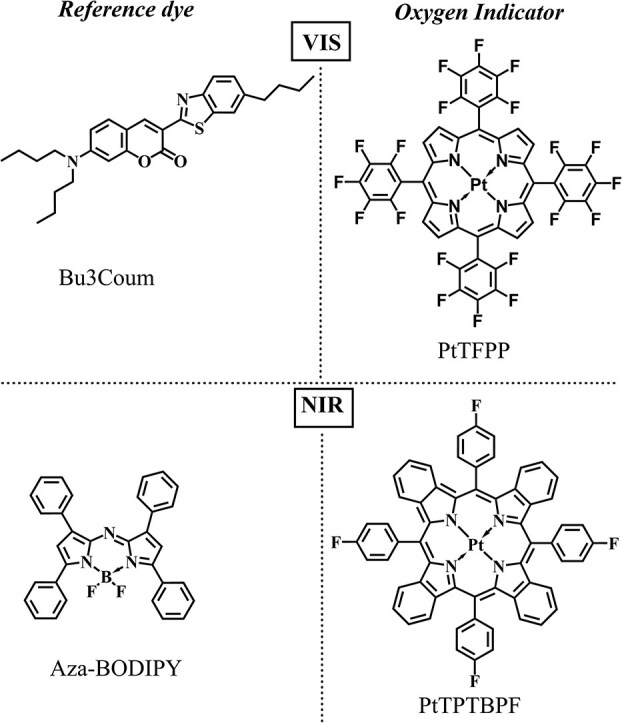
Chemical structures of the luminescent dyes used in this study for preparing the VIS and NIR sensing layers.

Two different semi-transparent sensors were tested in this study, referred to as VIS and NIR, which were prepared starting from the chemical “cocktail” recipes presented in Table 1 and using dyes with chemical structures indicated in Fig. 2. Here, the VIS sensor recipe relies on a commonly employed combination of a fluorescent coumarin antenna dye (either commercial Macrolex Yellow or a more lipophilic version Bu3Coum to prevent leaching out of the foil) and the red-emitting porphyrin-based O_2_ indicator PtTFPP [35]. The NIR recipe relies on the combination of the fluorescent aza-BODIPY reference dye and NIR benzoporphyrin indicator, the pair that was recently utilized in O_2_-sensing nanoparticles in combination with cell staining [36]. The NIR recipe has now been adopted for planar sensors whose optical compatibility with living bio-engineered fluorescent bacteria was tested in this study.

**Table 1:** Recipes for VIS and NIR sensing layers production, including suppliers or references for chemical purchase or in-house production. Bold font identifies labels of luminescent dyes used in the following.

Chemical	Supplier	VIS	NIR
**Aza-BODIPY**	In-house produced [37]	-	4 mg
**PtTPTBPF** (Platinum(II) *meso*-tetra (4-fluorophenyl) tetrabenzoporphyrin)	Frontier Scientific	-	2 mg
Polystyrene	Fisher Scientific	400 mg	400 mg
Chloroform	Sigma-Aldrich	5300 mg	5200 mg
**Bu3Coum** (Lipophilic coumarin dye)	In-house produced [38]	4 mg	-
**PtTFPP** (Platinum(II) meso-tetra(pentafluorophenyl)porphyrin)	Frontier Scientific	2 mg	-

Sensor “cocktails” were prepared in brown glass vials, wrapped in aluminum foil, and mixed overnight at 200 rpm. To improve sensor adhesion, glass microscopy slides (75 mm × 50 mm, Fisher Scientific) were ethanol-cleaned and silanized with chlorotrimethylsilane (Sigma-Aldrich). Then, ~200 μL of sensor “cocktail” was spread on each glass slide via knife-coating (BYK, Film width 4.5 in, Gap Clearance 2 mils) and the chloroform was allowed to evaporate in a fume hood under dark conditions. Absorbance spectra and emission spectra were acquired on Agilent Cary 60 UV–VIS Spectrophotometer and Florolog 3 spectrofluorometer (Horiba), respectively.

### Bacterial cultivation

GFP and mScarlet-I are among the most used and brightest fluorescent proteins available for genetically engineered microorganisms. These proteins present distinct excitation and emission peaks (https://www.fpbase.org/), which allows discriminate optically bacterial populations in a mixed community [31]. To test the compatibility of luminescent O_2_ sensors with fluorescent proteins, we used GFP *Pseudomonas putida* GB1 (Green *P. putida*) and mScarlet-I *Pseudomonas veronii* 1YdBTEX2 (Red *P. veronii*). Both strains were grown aerobically overnight in pure Luria Bertani broth (LB, Sigma Aldrich) in the dark in an orbital shaker at 180 rpm at 30°C. The overnight cultures were used to prepare the inoculum as detailed below, depending on the experiment performed. When needed for ease of reading, GFP and mScarlet-I signals are referred to as Green (G) and Red (R) signals.

### Sensor and fluorescent protein emission spectrum compatibility

To evaluate the compatibility between the sensor and the fluorescent protein emission spectra, a drop of overnight bacterial culture was deposited onto sensor-coated glass slides. The samples were kept in the dark for a few hours to reduce the water content of the bacterial incubation via evaporation until the sample was still wet but sufficiently immobilized on the glass surface to facilitate manipulation during the following measurements. Emission spectra of the fluorescent bacteria in combination with the sensors were then recorded using a spectrofluorometer (Florolog 3, Horiba). Anoxia was imposed during the measurements using a customized gas chamber compatible with the spectrofluorometer and controlling its atmosphere with a red-y smart gas controller from Vögtlin Instruments (Switzerland), injecting nitrogen (99.999% purity). Measurements were repeated at different excitation wavelengths corresponding to the absorbance maxima of the fluorescent proteins and the sensor. Compatibility was assessed by quantifying possible emission peak shifts and changes in luminescent signal detection caused by signal overlap. Emission spectra of mScarlet-I and GFP were also measured alone as a reference, using a similar procedure with an uncoated glass slide.

### Sensing microfluidic fabrication

**Figure 3:**
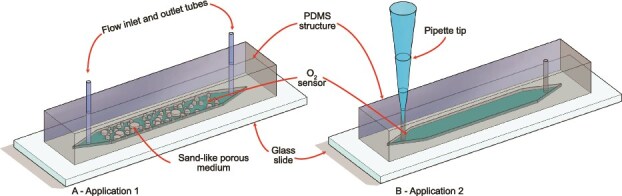
Schematics of sensing microfluidic devices used in applications 1 (a, Section Application 2: Single-cell detection) and 2 (b. Section Application 1: Multi-strain cell aggregates and self-induced O_2_ gradients).

The sensing microfluidic devices used in this study were fabricated by plasma bonding (Plasma Bonding Pen, ElveFlow) a polydimethylsiloxane (PDMS) structure (Sylgard 184 Silicone Elastomer mixed with 10 wt% curing agent; Dow Corning) onto a sensor-coated glass slide [27, 28]. The PDMS structures incorporated different microfluidic geometries depending on the target application (Fig. 3), which were produced by casting PDMS onto molds prepared using standard soft lithography (FlowJem). Inlet and outlet ports were created with a biopsy puncher (1.25 mm) on the PDMS structure before proceeding with plasma bonding. Because plasma bonding was only partially effective on sensor-coated surfaces, the sensors were manually trimmed to match the shape of the microfluidic devices, and excess material was peeled off to maximize direct contact between the PDMS and the exposed glass surface.

### Application

#### Fluorescence microscopy

The non-invasive simultaneous mapping of O_2_ concentrations and green and red signals can be performed using any fluorescence microscope equipped with a scientific black-and-white camera and a suitable set of objectives and cubic filters. In this work, we used a Zeiss AxioObserver Z1 inverted microscope equipped with a Photometrics CoolSnap HQ2 camera, a 20× (EC Plan Neofluar 20×, N.A. 0.5 Ph2) and 10× objectives (EC Plan Neofluar 10×, N.A. 0.3 Ph1). To map the fluorescent bacteria and the O_2_ sensor signal, four cubic filters (AHF) were installed. As the VIS sensor was not selected for the applications based on spectrofluorometry (see Section Sensor and fluorescent protein spectrum compatibility), we optimized the setup only for the NIR sensor dyes and fluorescent bacteria (see SI, Section S1, Table S1). Imaging strategy and processing, performed via Axiovision 4.8 software, Fiji ImageJ, and in-house produced MATLAB codes, depended on the application and are detailed in Section Results and SI (Section S4), where images are presented.

#### Application 1: Multi-strain cell aggregates and self-induced O_2_ gradients

This application was designed to rapidly promote bacterial aggregation by supplying abundant nutrients, O_2_, and a dense inoculum. The goal was to create localized anoxic microsites for testing luminescent sensors in detecting microscale microbially driven O_2_ gradients while tracking the spatiotemporal dynamics of fluorescent bacterial aggregates. To this end, we fabricated a microfluidic device replicating the porous structure of sandy sediments (Fig. 3a) that has previously shown to sustain microbial colonization and O_2_ gradient formation [4]. The device measured 27 mm × 5 mm × 50 μm. After PDMS degassing (20 min, vacuum desiccator), a bacterial suspension of 40% overnight Green *P. putida* and 60% overnight Red *P. veronii* was inoculated from the outlet port. The ratio accounted for strain-specific overnight culture OD_600_ values (1.38 and 0.92 for *P. putida* and *P. veronii*, respectively) to ensure initial comparable cell concentrations. Following 30 min for pore saturation and cell attachment, Tygon tubes were connected at both ends. The inlet tube was linked to a syringe pump (Harvard Apparatus, Pump 11 Elite) to deliver nutrient- and O_2_-rich LB (50% v/v) at 0.9 μL min^−1^ for 12 h. Spatial organization of bacteria and O_2_ was mapped every 2 h using four fluorescence channels (Green, Red, O_2_-sensitive dye, reference dye, see SI, Section S1) plus bright-field microscopy for total biomass (phase-contrast) and porous structure (bright-field). At each step, 80 images were acquired at 10× magnification (~1 μm/pixel) and stitched to reconstruct the entire porous space at high resolution.

#### Application 2: Single-cell detection

The fluorescent signal expressed by engineered bacteria, especially in the planktonic phase, can be much less intense than that of a dye. It is therefore important to test the suitability of O_2_-sensing microfluidic devices combined with engineered fluorescent bacteria to map single cells with changing O_2_ concentration. We used a straight-channel microfluidic device (27 × 5 × 0.05 mm; Fig. 3b) inoculated with Green *P. putida* and Red *P. veronii* (20× diluted overnight cultures in LB). After two hours of sedimentation on the sensor-coated glass surface, a 200 μL pipette tip was inserted in the inlet port, and Na₂SO₃ crystals were added that, dissolving into the medium, generated an O_2_ gradient chemically. Following ~20 minutes of equilibration, five fields along the channel were imaged at 20× using green, red, and the sensor’s reference/emission channels.

## Results

### Sensor and fluorescent protein spectrum compatibility

**Figure 4:**
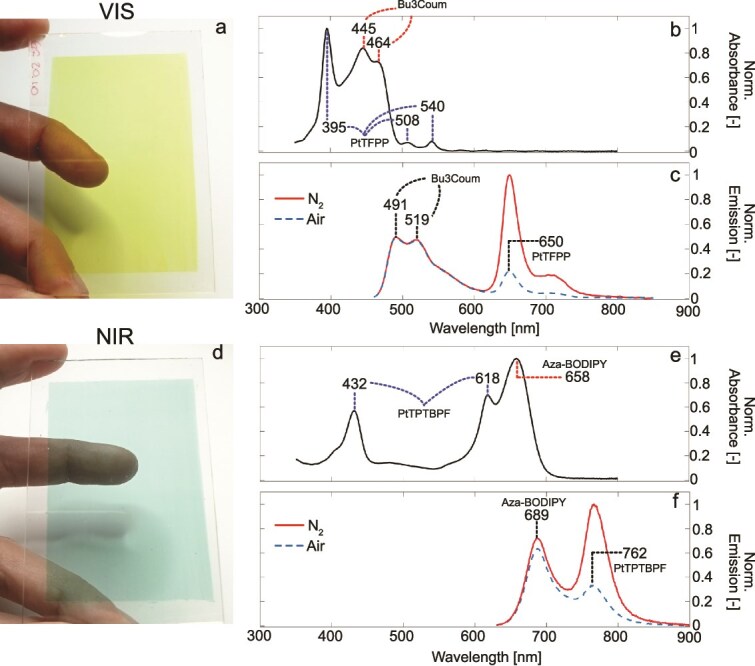
Pictures of the sensing layers (a and d) after solvent evaporation, screen-printed on a microscope glass slide (75 mm ×50 mm). Edges of the sensing layer are trimmed and manually removed for a regular rectangular shape. Absorbance and emission spectra of VIS (b, c) and NIR sensing layers (e,f). Emission spectrum is reported under a nitrogen (N_2_) atmosphere (ie, 0% v/v O_2_) and air (ie, 21% v/v O_2_) with excitation wavelength at 450 nm and 615 nm for VIS and NIR sensors, respectively. All emission and absorbance spectra are normalized to the highest peak value. Peak positions and values are also reported in Tables S3 and S4 in SI, Section S2.

The produced sensing layer was about ~5 μm in thickness (Fig. 4a and d) measured with Mahr Extramess 2001. The intrinsic response time of the sensing layer was estimated to fall between 2–5 s based on the diffusion timescale of O_2_ in the polystyrene [39] and additional experimental tests of fast anoxic-oxic transitions under well-stirred conditions (see SI, Section S2). However, in practical applications, previous studies on similar sensing layers showed that response time can be considerably longer, up to 60 s [40]. Despite the relatively high uncertainty, this response time is much smaller than the image acquisition frequency (every 2 hours) and is suitable for most microbial ecology dynamics, which typically present timescales between minutes and hours.

As both VIS and NIR sensors are composed of a mixture of an O_2_-sensitive dye and a reference dye, the absorbance spectra (Fig. 4b and e) were the result of the superimposition of the absorbances of the two dyes composing each material. As such, these spectra present multiple absorption peaks and were used to identify the optimized excitation wavelength, ie, 450 nm and 615 nm for VIS and NIR sensors, respectively. Similarly, the emission spectra are also the superimposition of two luminescent signals with distinguishable peaks (Fig. 4c and f). After calibration (see SI Section S2, Figure S1 and Table S2), O_2_ concentration can be estimated via a ratiometric approach, ie, from the ratio between the O_2_-sensitive and the reference signals, which compensates for the variations, e.g., due to inhomogeneous illumination or detector sensitivity.

**Figure 5:**
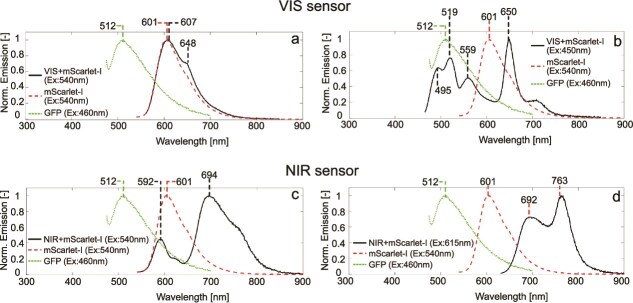
Emission spectra for mScarlet-I *P. Veronii* combined with VIS (a,b) and NIR (c,d) sensors generated by different excitation wavelengths under anoxic atmosphere. Dashed vertical lines highlight the position of emission peaks identified in each spectrum. As a reference, we reported in each plot the emission spectra of mScarlet-I (red dashed line) and GFP (green dotted line) measured without sensors excited at 540 nm and 460 nm, respectively. Peak positions and values are also reported in Tables S3 and S4 in SI, Section S3.

Spectrofluorometry was used to record emission spectra from combinations of mScarlet-I *P. veronii* and the VIS or NIR sensors under different excitation conditions. Excitation at 540 nm, chosen to optimize bacterial fluorescence, partially excited the VIS sensor (Table S3) due to the absorption maximum of PtTFPP (so-called Q band) located at this wavelength (Fig. 4b). As a result, sensor emission was not completely suppressed (Fig. 5a), altering the mScarlet-I signal at 648 nm, corresponding to PtTFPP (O_2_-sensing dye) emission peak. When excited at 450 nm, ie, the excitation wavelength that is optimal for the VIS sensor emission, mScarlet-I was partially excited as well (Fig. 5b). As a result, the mScarlet-I emission interfered with VIS emission when mScarlet-I and VIS emissions were measured as a combined spectrum at 450 nm excitation wavelength. Indeed, the overlap with the mScarlet-I signal unevenly shifted up the Bu3Coum dye spectrum, altering the relative intensities of the 495 and 519 nm peaks compared to the emission of the sensing layer without any fluorescent background (Fig. 4c). The presence of mScarlet-I distorted the O_2_-sensitive/reference dye peak ratio: under anoxic conditions, the expected peak ratio (650 nm/519 nm) of 2.08 (from Fig. 4c, Table S4, SI, Section S3) dropped to 1.32 (from Fig. 5b, Table S4, SI, Section S3). Moreover, as expected from GFP spectral properties (https://www.fpbase.org/) and confirmed by direct measurements of GFP emission spectrum (Fig. 5b, further discussed in SI, Section S3, Figure S2), GFP excitation and emission wavelengths completely overlap with those of Bu₃Coum, making the VIS sensor and GFP fluorescence indistinguishable.

Like the VIS sensor, the NIR sensor emission was not fully suppressed at 540 nm (Fig. 5c), despite low absorbance at this wavelength (Fig. 4e). However, the overlap of the signals from mScarlet-I and the NIR sensor was negligible. The mScarlet-I emission peak remained well separated from sensor emission, and its wavelength matched the characteristic one (603 nm). Moreover, the fluorescent bacteria did not alter the emission spectrum of the NIR sensor. The reference (693 nm) to O_2_-sensitive (763 nm) dye ratio under anoxic conditions was identical with or without mScarlet-I bacteria (1.37; Fig. 4f  *versus*  Fig. 5d, Table S4, SI, Section S3). The GFP emission spectrum is in a shorter wavelength range compared to mScarlet-I (see SI Section S3, Fig. S2), making the GFP signal also clearly distinct from the NIR sensor one. Overall, the NIR sensor provided an interference-free signal with fluorescent bacteria.

### Maps of fluorescent populations and O_2_ concentration

**Figure 6:**
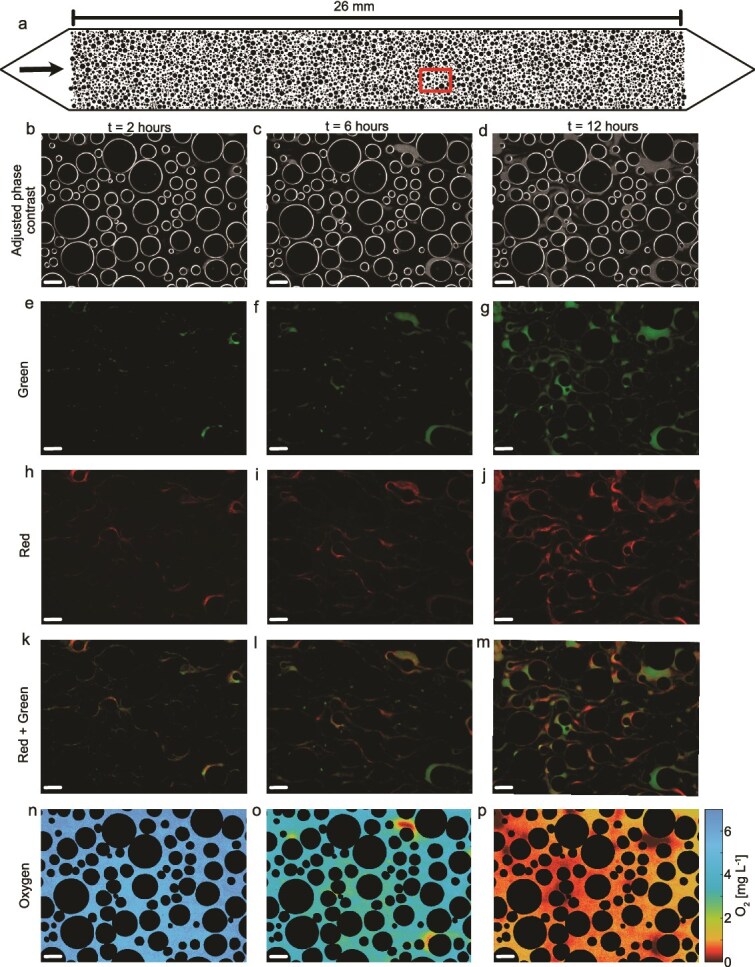
a) Porous medium geometry mimicking a sandy structure engraved into the O_2_-sensing microfluidic device. Black arrow: Flow direction; rectangular box: Region shown in panels b–p. (b–d) Phase contrast images showing biomass and pore structure at 2, 6, and 12 h after flow onset. e–g) Signal of *P. Putida* at 2, 6, and 12 h. (h–j) signal of *P. Veronii* at 2, 6, and 12 h. k–m) overlays of GFP (*P. Putida*) and mScarlett-I (*P. Veronii*) signals. N–P) O_2_ concentration maps at 2, 6, and 12 h (ratiometric approach; SI Section S2). Scale bars are 100 μm-long. Full-porous landscape maps and image processing details are provided in SI (Section S4, Figure S3).

Time-lapse microscopy enabled microscale mapping of the spatiotemporal dynamics of both strain-specific biomass (~1 μm pixel^−1^) and O_2_ concentration (chemical mapping resolution ~10–15 μm [41], see SI, Section S4 for further details) across the entire porous structure (Fig. 6, full maps in SI, Section S4). Biomass progressively colonized the pore space (Fig. 6b-d), forming dense aggregates. Fluorescent signals from the two strains were frequently detected at the same locations (Pearson correlation coefficient > 0.5; see Table S5 in SI, Section S5), as illustrated in Fig. 6k-m. Nevertheless, high-resolution spatiotemporal distributions of *P. putida* and *P. veronii* could be resolved independently. Consistent with previous studies demonstrating the compatibility of green and red fluorescent proteins [31], these results indicate that the NIR sensor did not interfere with the detection of either fluorescent signal. Cross-comparison of all maps further shows that, although correlated, the spatiotemporal distributions of *P. putida, P. veronii*, and O_2_ concentration exhibited distinct local patterns. These point-by-point differences in signal intensity confirm results obtained by spectrofluorometry and suggest that the observed spatial correlations arise from biogeochemical dynamics rather than from signal detection artefacts.

**Figure 7:**
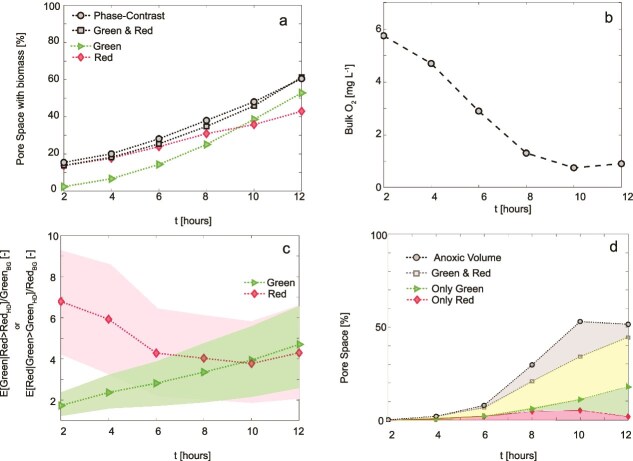
a) Comparison of total pore space occupied by biomass estimated from phase-contrast images (circles) and fluorescence-based maps (squares). Contributions of *P. Putida* (GFP; triangles) and *P. Veronii* (mScarlet-I; diamonds) are shown separately. b) Temporal evolution of bulk O_2_ concentration in the porous medium. c) Mean fluorescence intensity of *P. Putida* measured in regions of high *P. Veronii* density (triangles) and vice versa (diamonds); values are normalized to the corresponding background signals. d) Fraction of pore space under anoxic conditions (O_2_ < 0.42 mg L^−1^), partitioned into four categories: (i) colonized only by *P. Veronii* (diamonds), (ii) colonized only by *P. Putida* (triangles), (iii) co-colonized by both strains (squares), and (iv) uncolonized (circles).

The fraction of pore space colonized by biomass was quantified using both phase-contrast and fluorescent-protein images and compared (Fig. 7a; details in SI, Section S6). This comparison shows that, despite the presence of the sensor, fluorescent-protein signals provided a reliable quantification of biomass colonization, closely matching trends derived from phase-contrast images.

The use of fluorescent bacterial signals enabled discrimination of the pore space occupied by the two different strains (Fig. 7a). Both strains exhibited a continuous increase in biomass over time. Although *P. putida* initially occupied a smaller fraction of the pore space than *P. veronii*, it displayed a faster colonization rate. Bulk O_2_ concentration (Fig. 7b) exhibited progressive decreasing with time, until approximately 1 mg L^−1^, consistently with a large portion of pore space (>50%) colonized by O_2_-consuming biomass.

### In situ spatial analyses

Our microfluidic platform provides simultaneous, full optical access to the spatial distributions of multiple microbial strains and O_2_ concentration, enabling in situ spatial correlation between microbial organization and local chemical conditions. A wide range of analyses could be performed, with metrics targeting different ecological questions. Here, we present selected examples to illustrate the potential of this methodology.

Although coexisting within the same aggregates, the relative spatial distributions of the two strains were heterogeneous and evolved with time (Fig. 6k-l). To characterize the dynamics of *P. veronii* and *P. putida* organization, we defined two fluorescence thresholds for each strain: (i) a background threshold discriminating against bacterial signal from background (Green_BG_, Red_BG_), and (ii) a threshold separating low-density from high-density biomass regions (Green_HD_, Red_HD_; see SI, Section S6, Figure S4). Spatial relationships between low- and high-density regions of the two strains were analyzed using conditional probability density functions (reported in SI, Section S7, Figure S5).

Initially, aggregates with high *P. putida* density were also characterized by high *P. veronii* density, with mean red fluorescence values approximately seven times higher than the red background signal (Red_BG_, Fig. 7c). In contrast, regions dominated by high *P. veronii* density exhibited mean green fluorescence values comparable to the green background (Green_bg_). Thus, dense colonies of only *P. veronii* were detected, whereas dense *P. putida* colonies were consistently co-located with dense *P. veronii* regions. Over time, this asymmetry progressively diminished: *P. putida* accumulated within *P. veronii*-dominated clusters, while *P. veronii* density decreased within *P. putida*-rich regions. Eventually, the two strains converged toward similar spatial distributions, such that regions dominated by one strain were moderately enriched with the other.

Regions dominated by green *P. putida* (Fig. 6e-g) appeared more frequently organized into rounded aggregates. In contrast, aggregates dominated by red *P. veronii* frequently exhibited elongated morphologies aligned with streamlines or forming coatings on grains. Using morphological analysis, we confirmed that high-density *P. putida* aggregates showed higher circularity than high-density *P. veronii* aggregates (details in SI, Section S8, Table S6).

Oxygen maps (Fig. 6n-p) revealed the progressive development of O_2_ gradients. These maps enable estimation of the local O_2_ concentrations experienced by microbial cells and the identification of potential hotspots of phenotypic differentiation in response to self-induced O_2_ depletion. For example, we quantified the anoxic volume as the pore space with O_2_ < 0.42 mg L^−1^ (Fig. 7d), corresponding to the estimated detection limit of the sensing layer in the selected system (SI, Section S2). By combining O_2_ maps with strain-specific fluorescence signals, we quantified the fraction of anoxic pore space colonized by *P. putida* and *P. veronii*, respectively. Both strains were often co-located within anoxic regions (Fig. 7d). However, regions characterized by anoxic conditions and colonization by only *P. putida* strain progressively emerged. Instead, the fraction occupied exclusively by *P. veronii* peaked at approximately 5% after 10 h and became negligible by 12 h. Finally, a non-negligible fraction of uncolonized anoxic pore space existed, particularly during the final 4 h of the experiment.

In application 2, we demonstrate that individual fluorescently tagged cells can be resolved under varying O_2_ concentrations, provided that appropriate magnification is used (SI, Section S9, Figure S6).

## Discussion

The combined use of microfluidics, fluorescent optical sensing, and fluorescent bacteria represents a sought-after methodological advance in microbial ecology. Real-time visualization of microbial self-organization and chemical gradients within confined environments is essential to elucidate microscale coupled microbial and chemical dynamics that regulate the emergence of ecological functions in microbial communities. This study demonstrates that, through careful selection of fluorescent protein spectral properties and by leveraging the latest generation of optical sensing technologies, this methodological advance is achievable.

Currently, the most widespread optical O_2_-imaging technologies, including commercial solutions, rely on VIS-based sensor formulations. The use of PtTFPP as a sensing dye is well established in environmental and medical applications (eg, [26–30, 32, 42–44]). Its broad adoption stems from key properties such as high photostability, reversible O_2_ quenching, and compatibility with polymeric matrices, which together ensure versatility, reliability, and repeatability of measurements. The NIR sensor formulation proposed here shares these fundamental characteristics and therefore offers comparable versatility across a similar range of applications.

Our explicit validation of spectral interactions demonstrates that VIS sensor recipe is incompatible with fluorescent proteins commonly used in bioengineering. Although fluorescent protein signals are typically weaker than the luminescent emission of sensor dyes, dense fluorescent cell aggregates can nonetheless induce measurable shifts in emission peaks and alter ratiometric values, thereby biasing VIS sensors. In contrast, the NIR sensor exhibited the absence of spectral interference, with distinguishable emission peaks and stable ratiometric responses regardless of the presence of fluorescent bacteria. The spectrofluorometric comparison between VIS and NIR sensors presented here, based on peak shifts and ratiometric distortions, defines a quantitative and reproducible framework to guide the design of sensing microfluidic experiments in microbial ecology and to evaluate sensor–fluorophore compatibility. Our assessment focused on the ratiometric approach. We note, however, that an alternative method based on phosphorescence decay time can also be used to estimate O_2_ concentration and is compatible with the setup proposed here. While this approach offers advantages, particularly in reducing potential interferences and minimizing sensing layer complexity (reference dye is not needed), it requires specialized imaging equipment that is not yet widely available in standard imaging facilities. As a result, it is generally less accessible and more difficult to implement compared to the ratiometric approach adopted in this study.

We further demonstrated that the detection of single cells and multi-strain microbial aggregates is not compromised by the presence of the NIR sensor. Fluorescent labeling is a widespread technique in microfluidics, possessing the advantages of facilitating visualization in complex matrices and high sensitivity [45]. In this context, NIR-based sensing in microfluidic devices offers clear advantages, particularly in systems involving biomineralization or microbially induced mineral dissolution. Beyond their spectral compatibility with fluorescent bacteria, using far-red emitting probes is well aligned with emerging trends in biological imaging, where longer wavelengths are increasingly favored due to reduced light scattering, lower autofluorescence, and improved multiplexing capability [38]. In addition, shifting sensor operation to the NIR spectral range minimizes undesirable side effects such as fluorescent protein photobleaching, expected with VIS sensors whose excitation wavelength partially overlaps with the absorbance spectra of fluorescent proteins. The NIR sensor configuration effectively avoids this overlap, further reinforcing its suitability for long-term, high-resolution microbial ecology experiments.

The primary aim of this study was to demonstrate the potential of a sensing microfluidic platform enabling simultaneous visualization of microbial organization and self-induced O_2_ gradients. Nevertheless, the spatial analyses presented here highlighted some patterns that can be interpreted considering existing knowledge on microbial behavior in confined, flow-through porous environments.

The two bacterial strains exhibit contrasting shapes, ie, elongated *P. veronii* and rounded *P. putida* (see Section S9). Previous studies showed that cell morphology strongly influences attachment and transport in porous media, even in the absence of functional or metabolic differentiation [24]. Cells with different shapes experience distinct hydrodynamic torques, resulting in colonies of elongated bacteria preferentially aligned with the flow direction. Our observations are consistent with this framework: *P. veronii* preferentially formed more elongated structures, with lower circularity, oriented along streamlines. *P. putida* tended to organize into more compact, rounded aggregates in pore-space bulges.

At 2 hours from the onset of the flow (the earliest time point imaged for technical reasons), pore space colonization was dominated by *P. veronii* (~20%), representing almost the totality of the colonizing biomass identified, despite similar cell concentrations being present in the inoculum. This early dominance is consistent with the more efficient attachment of elongated, motile cells under flowing conditions in confined environments, as previously reported [11, 24]. In contrast, according to batch cultures (SI, Section S10, Figure S7 and Table S7), *P. putida* may exhibit a shorter lag phase than *P. veronii*. Recent studies have shown that, following environmental perturbations, strains with faster recovery (or shorter lag phase) tend to dominate population dynamics under continuous flow [23]. Over time, this growth advantage could have contributed to the progressive accumulation of *P. putida* within regions initially dominated by *P. veronii*, thereby reducing the early uneven pore-space occupancy.

The combined effects of differential attachment efficiency and growth rate are also consistent with the observation that high-density aggregates composed exclusively of *P. veronii* were present at early stages. In contrast, dense *P. putida* clusters were always co-located with *P. veronii. P. veronii* clusters formed preferential attachment sites along streamlines that subsequently enhanced the retention of planktonic *P. putida* cells. Once established, *P. putida* would then progressively increase its contribution to the aggregate biomass. Such interpretations are merely based on the shape and growth rate of the cells during the exponential growth phase. We cannot exclude the possibility that indirect feedback, such as that observed in [15], occur, but detailed investigations are beyond the scope of this work.

The formation of sharp O_2_ gradients and extended anoxic microsites is consistent with previous observations in dense microbial aggregates in similar conditions [28]. Both strains were located in anoxic regions with no clear evidence of O_2_-driven spatial segregation, consistent with both strains respiring only aerobically in the chosen medium. Although previous studies observed a microbial response to O_2_ depletion within less than two hours [22], it is possible that the experiment time horizon was insufficient to observe a reorganization after the onset of the anoxic microsites. Flow-induced shear may promote the formation of compact, EPS-rich biofilms [46] that might resist structural reorganization. Still, the increasing dominance of *P. putida* in anoxic regions at later times may reflect its tendency to form more rounded colonies, which limit O_2_ supply within the colony core compared to elongated colonies, favoring the onset of anoxic conditions. [28].

Altogether, this study demonstrates the potential of near-infrared (NIR) sensing microfluidic approaches to elucidate microbial community ecology and organization. We argue that this potential can be further expanded by harnessing ongoing advances in microfabrication, bioengineering, and optical and analytical sensing technologies.

First, the proposed approach is compatible with all fluorescent proteins whose emission peaks at wavelengths shorter than that of mScarlet-I (<562 nm). The wide range of fluorescent proteins available provides flexibility in designing multi-strain communities. For example, in the SI (Section S11, Figure S8), we describe a combination of four fluorescent proteins compatible with NIR sensor emission, enabling real-time in vivo visualization of a four-strain community. Furthermore, deep-learning–based image analysis could increase the number of distinguishable strains by exploiting additional features such as cell morphology or size, even when emission spectra partially overlap [47]. The semi-transparency of the sensor also ensures compatibility with optical and fluorescence-based techniques widely used in microbial ecology, like fluorescent stains (eg, DAPI, live–dead staining) [46], and emerging approaches of spatial omics [48]. Particularly promising is the possibility to first map O_2_ distributions in real time and subsequently apply fluorescence in situ hybridization (FISH, [15]) on the same (fixed) sample, providing unique insights into a direct correlation between local chemical microenvironments and the taxonomic identity of microorganisms.

Beyond O_2_, additional physicochemical parameters can already be mapped using NIR sensors, including potassium concentration, pH, and temperature [49–51], key factors controlling microbial dynamics [1, 10, 52]. Because our methodology relies on epifluorescence microscopy, excitation and detection occur from the same side of the sample, making it inherently compatible with opaque geomaterials such as rocks and sediments, in principle independent of sample thickness. However, the integration of natural materials into microfluidic platforms remains largely exploratory, with only limited demonstrations for consolidated substrates, eg, rocks [6, 53, 54], and no established protocols for unconsolidated sediments. In this context, current limitations are primarily related to fabrication challenges rather than sensing performance, and further developments are needed to fully realize these applications.

Finally, when properly designed, microfluidic platforms allow different microscopic and spectroscopic in situ inspections, helping to, e.g., characterize 3D biofilm structures and anabolic patterns, biomineralization pathways, and organic carbon-microbe aggregates [55–58]. The use of such techniques on the proposed sensing platform has the potential to deliver an integrated description of microbial-mineral and organic particulate interactions in addition to chemical and microbial ones. In conclusion, this approach provides a cost-effective, customizable, and broadly accessible platform for investigating how microbial communities shape and adapt to their microenvironments, and how self-organization mediates responses to chemical and physical stresses in confined environments.

Although the NIR sensor demonstrated compatibility with multiple bio-engineered fluorescent bacteria, it does not necessarily outperform conventional VIS-based sensors across other performance metrics, such as response time, spatial resolution, detection limits, susceptibility to interferences, or potential toxicity to microbial life. Both VIS and NIR sensors rely on the same detection principle and on the indicators of the same class (platinum(II) porphyrins) and therefore share many of the same advantages and limitations. For instance, very similar phosphorescence decay times of PtTFPP and PtTPTBPF result in comparable sensitivity and limits of detection, whereas the dynamic response is determined solely by the thickness of the sensing layer due to an identical polymeric matrix. More broadly, optical chemical sensing remains a relatively recent and rapidly evolving technology. While it offers considerable flexibility and versatility in design [26], sensing layeror fabrication, integration into microfluidic platforms, and optical readout are not yet standardized and remain highly dependent on: i) the optical configuration (eg, objective, camera, illumination source, and filters); ii) sensing layer properties (eg, thickness and matrix composition); and iii) image processing workflows.

As a result, key performance metrics such as spatial resolution and detection limits are not intrinsic properties of the sensing chemistry alone, but emerge from the full experimental setup. For instance, spatial resolution is constrained by O_2_ diffusion within the sensing layer, waveguiding of the luminescent signal in it [41], and signal processing, typically resulting in effective resolutions significantly larger than the optical pixel size (SI, Section S4). Similarly, detection limits depend on the signal-to-noise ratio and the local sensitivity of the calibration curve, and therefore vary with experimental conditions (SI, Section S2).

Additional limitations remain insufficiently characterized. For example, the potential impact of redox-active metabolites on luminescent signals [59], the interference from other non-polar chemicals, as well as the possible toxicity of sensing dyes or polymer matrices. While the use of polymer-embedded dyes is expected to significantly reduce direct interactions with cells [44] and limit leaching, comprehensive evaluations of biocompatibility and long-term stability are still lacking. As the complete elimination of sources of uncertainty is likely impractical, the use of sensing microfluidic devices could be complemented by approaches designed for uncertainty quantification and propagation to metrics of interest [60, 61].

Overall, these considerations highlight that, while luminescent sensing layers provide powerful opportunities for chemical imaging in microbial systems, careful experimental design and critical interpretation of the data remain essential. Further work toward standardized protocols and systematic characterization of sensor performance will be important to fully exploit the potential of these techniques.

## Supplementary Material

SI_Revised_V2_ycag146

## Data Availability

The datasets generated and/or analyzed during the current study are available from the corresponding author on reasonable request.
