# Supplementary material for: Simultaneous real-time imaging of oxygen gradients and in vivo microbial community spatial organization in confined environments
Source: ISME Commun. 2026 May 29;6(1):ycag146. doi: 10.1093/ismeco/ycag146 (PMC13298642; doi:10.1093/ismeco/ycag146)
Supplement: SI_Revised_V2_ycag146 [file si_revised_v2_ycag146.docx]

**Supplementary Information for “Simultaneous real-time imaging of oxygen gradients and *in vivo* microbial community spatial organization in confined environments.”**

Giulia Ceriotti^1^, Sergey M. Borisov ^2^

^1^ Institute of Earth Surface Dynamics, Faculty of Geoscience and Environment, University of Lausanne, 1012 Lausanne, Switzerland

^2^Institute of Analytical Chemistry and Food Chemistry, Faculty of Technical Chemistry, Chemical and Process Engineering and Biotechnology, Graz University of Technology, 8010 Graz, Austria

# Cubic filters and exposure times

Table S 1 Properties of cubic filters used to map GFP, mScarlet-I, PtTPTBPF, and Aza-BODIPY signals and the chosen exposure time for each channel.

| Fluorescent signal | Excitation Filter | Emission Filter | Beamsplitter | Exposure time |
| --- | --- | --- | --- | --- |
| GFP *P. putida* | 469/35 nm | 525/39 nm | 497 nm | 200 ms |
| mScarlet-I *P. veronii* | 540/25 nm | 605/55 nm | 565 nm | 500 ms |
| PtTPTBPF | 615/45 nm | 775/50 nm | 683 nm | 3 ms |
| Aza-BODIPY | 628/40nm | 692/40 | 660 nm | 3 ms |

# Sensor response characterization, calibration and detection limit estimation

## Calibration and detection limit estimation

The approach we propose is designed to be reproducible, using this study as a reference, and consists of three steps:

- **STEP 1** **Sensor Characterization:** The response of the sensing layer was characterized to determine the shape of the calibration curve using a spectrofluorometric approach.
- **STEP 2 Sensor Calibration**: Preserving the shape of the calibration curve determined in step 1, we applied a two-point calibration protocol to adjust the sensing layer response to the specific optical setup of the microscope-camera combination used for the microfluidic experiment.
- **STEP 3 Detection limit identification**: Using the calibration curve and processing the images collected for the calibration is possible to define the detection limit of the sensing layer for the custom system used for the microfluidic experiment.

**STEP 1**: To characterize NIR sensor response to oxygen (O_2_), a sensing layer was fixed in a home-made flow-through chamber and exposed to different O_2_ partial pressures (values in Figure S 1). The atmosphere was controlled with red-y smart gas controllers from Vögtlin Instruments (Switzerland) by mixing nitrogen (99.999% purity) and compressed air. The luminescence spectra were acquired on a Fluorolog 3 spectrofluorometer (Horiba), imposing an excitation wavelength of 615 nm. The sensing layer response was found to attain a bi-exponential curve (Figure S 1) with the following expression:

$$\frac{I_{772}}{I_{691}}=A_{1}e^{- \frac{p_{O2}}{\tau_{1}}}+A_{2}e^{- \frac{p_{O2}}{\tau_{2}}}+q$$

where *I_772_* and *I_691_* are the two peaks of the sensor emission spectrum at 772 nm and 691 nm, respectively; *A_1_* and *A_2_* are pre-exponential factors, and *q* is a vertical shift fitting parameter; finally, *τ_1_* and *τ_2_* are time constants (values in Table S 2). If O_2_ concentration is expressed as mg L^-1^, *τ_1_* and *τ_2_* can be re-estimated, accounting for the change in units, and resulted in *τ_1_* = 0.896 mg L^-1^ and *τ_2_* = 4.75 mg L^-1^.


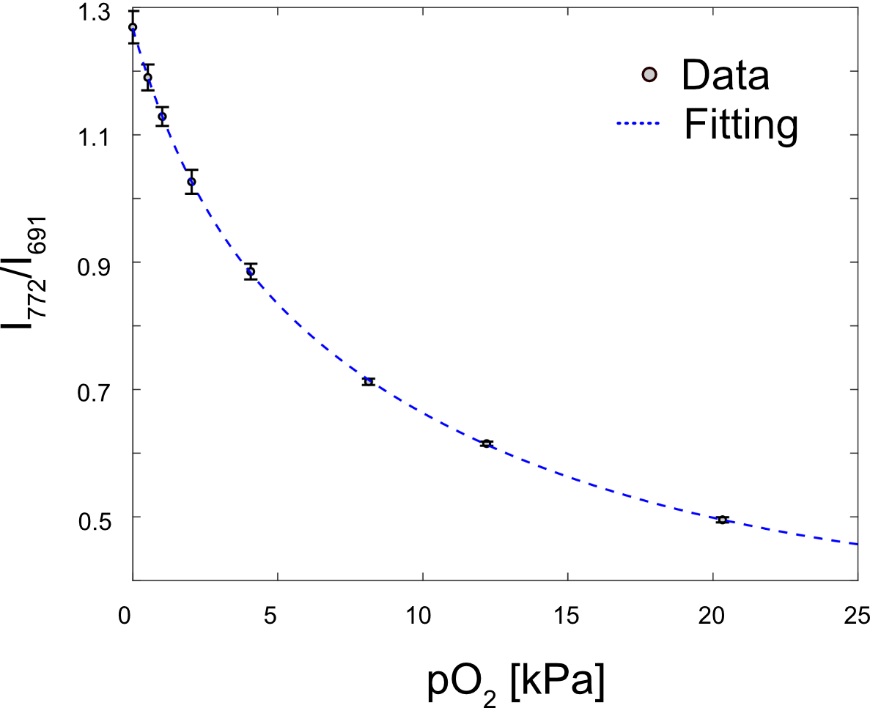


Figure S 1 Calibration data and the bi-exponential fit at 23 °C, i.e., the same temperature as microfluidic applications presented in this work. Vertical bar quantifies the standard deviation over three replicates of the measurements.

Table S 2 : Fitting parameters of the bi-exponential curve describing sensor response to increasing concentration of O_2_.

| Parameter | Symbol | Value |
| --- | --- | --- |
| Pre-exponential factor, exponential function 1 | *A_1_* | 0.23 [-] |
| Pre-exponential factor, exponential function 2 | *A_2_* | 0.66 [-] |
| Time constant, exponential function 1 | *τ_1_* | 2.22 [kPa] |
| Time constant, exponential function 2 | *τ_2_* | 11.75 [kPa] |
| Vertical shift fitting parameter | *q* | 0.38 [-] |

**STEP 2:** The bi-exponential curve is then used to calibrate the NIR sensing layer used for microfluidic experiments, accounting for the given microscope setup and optical system. The physics of quenching depends on the sensor physico-chemical properties, meaning that the bi-exponential behavior is preserved regardless of the optical system chosen, assuming no optical distortion. We performed a two-point calibration procedure to estimate pre-exponential factors (*A_1_* and *A_0_*) and vertical shift (*q*) to account for the impact of the optical and detection system used during the microfluidic experiment. An air-saturated solution was obtained by shaking and exposing to air 10 mL of 50%v/v LB medium three times. Concentration of O_2_ in the air-saturated medium was measured with the TROX430 sensor, PyroScience, and was equal to 7.1 mg L^-1^. An anoxic calibration solution (O_2_ concentration = 0 mg L^-1^, verified with TROX430) was prepared by mixing 10 mL of 50% v/v LB medium with 100 mg of sodium sulfite (Na_2_SO_3_, Sigma Aldrich). A drop of each calibration solution was deposited onto the O_2_ planar sensor in different locations. Images of the sensor luminescent signals were collected in 10 different locations in the center of the drop to avoid boundary effects and post-processed to obtain the values of the ratio *R*, i.e., the intensity of the O_2_-sensitive dye signal over that of the reference dye. Calibration curve parameters were estimated using MATLAB Curve Fitting App, which led to the following calibration curve:

$$R=\frac{I_{O2}}{I_{REF}}=0.472e^{-\frac{O_{2}[mg L^{-1}]}{0.896}}+0.768e^{-\frac{O_{2}[mg L^{-1}]}{4.75}}+0.948$$

where O_2_ is the concentration of dissolved O_2_ in the liquid, *I_O2_* and *I_REF_* are the luminescent signals of the O_2_-sensitive dye and the reference dye using the set of cubic filters and the exposure time reported in Table S 1.

**STEP 3:** Following standard IUPAC recommendations [1], the detection limit (*DL*) was estimated based on the signal-to-noise ratio and the local sensitivity of the calibration curve:

$$DL= \frac{3\sigma}{S}$$

where *σ* is the standard deviation of the signal, and *S* is the slope of the calibration curve. Given the nonlinear (bi-exponential) response of the sensor, the slope was evaluated in the region of interest, at low O_2_concentrations. The noise was estimated from O_2_ maps acquired during calibration under anoxic conditions as the standard deviation of the $I_{O_{2}}/I_{REF}$ratio. For consistency, the same processing pipeline was applied to these images as for O_2_ maps present in Section S4, i.e., a Gaussian filter is applied with a characteristic size of 5 μm. Based on this approach, we obtained a detection limit of 0.42 mg L⁻¹.

When replicating the methodology proposed in this manuscript, the only needed steps are **STEP 2** and **STEP 3**, i.e., a two-point calibration procedure, consistent with the standard practice also for commercial sensors routinely done in laboratory settings, and the same image processing pipeline chosen for the application.

## Sensor response time

The intrinsic response time of the sensing layer is primarily governed by oxygen diffusion within the polymer matrix. It therefore depends strongly on sensing layer thickness, polymer composition, and hydrodynamic conditions at the sensor–fluid interface. In the present study, the sensing layer thickness was approximately ~5 μm. Based on reported diffusion coefficients of oxygen in polystyrene (0.8–1.5 × 10⁻⁷ cm² s⁻¹ at 22–23 °C [7]), the characteristic diffusion timescale within the sensing layer itself is expected to be on the order of a few seconds.

To experimentally assess the intrinsic response of the sensing formulation, additional measurements were performed using a FireSting optical fiber system. The sensing layer was attached to the optical fiber. Excitation was performed at 625 nm and the integrated emission above 720 nm was detected. The sensing layer was alternately immersed in air-saturated water and in a stirred sodium sulfite solution (5 wt%) to impose rapid oxic/anoxic transitions while minimizing boundary layer effects. Under these well-stirred conditions, measured t_95_ response times were approximately 2 s for transitions from anoxic to oxic conditions and approximately 4.5 s for the reverse direction.

These measurements represent the intrinsic response time of the sensing layer under optimized transport conditions. In practical planar imaging applications, effective response times are generally longer due to additional contributions from oxygen transport across the sensor–fluid interface, local hydrodynamic conditions, boundary layer formation, and image acquisition and processing. Previous works with similar sensors suggested response times in planar optode systems to range between a few seconds and tens of seconds, depending on the experimental configuration, with a maximum of 60 s [8]. Nevertheless, these timescales remain substantially shorter than the temporal resolution used in the present study (hours) and are therefore fully suitable for investigating microbial ecological dynamics in the proposed setup and for many other microbial ecological dynamics.

# Summary of adsorption and emission peak positions and values

For the sake of clarity, Figure S2 reports the normalized emission spectrum of GPF – *P. putida* using an excitation wavelength of 460 nm. The resulting emission spectrum aligns with databases of reference (<https://www.fpbase.org/>). The GFP signal is compared to the VIS and NIR sensor emission (measured under anoxic conditions) using optimized excitation wavelengths to highlight: i) the overlapping of GFP and VIS signal, and ii) the clearly distinct peaks of NIR sensor and GFP. It is worth noting that the optimized excitation wavelengths for GPF and VIS sensors are very close, meaning that exciting the VIS sensor also excites the GFP protein and *vice versa*.


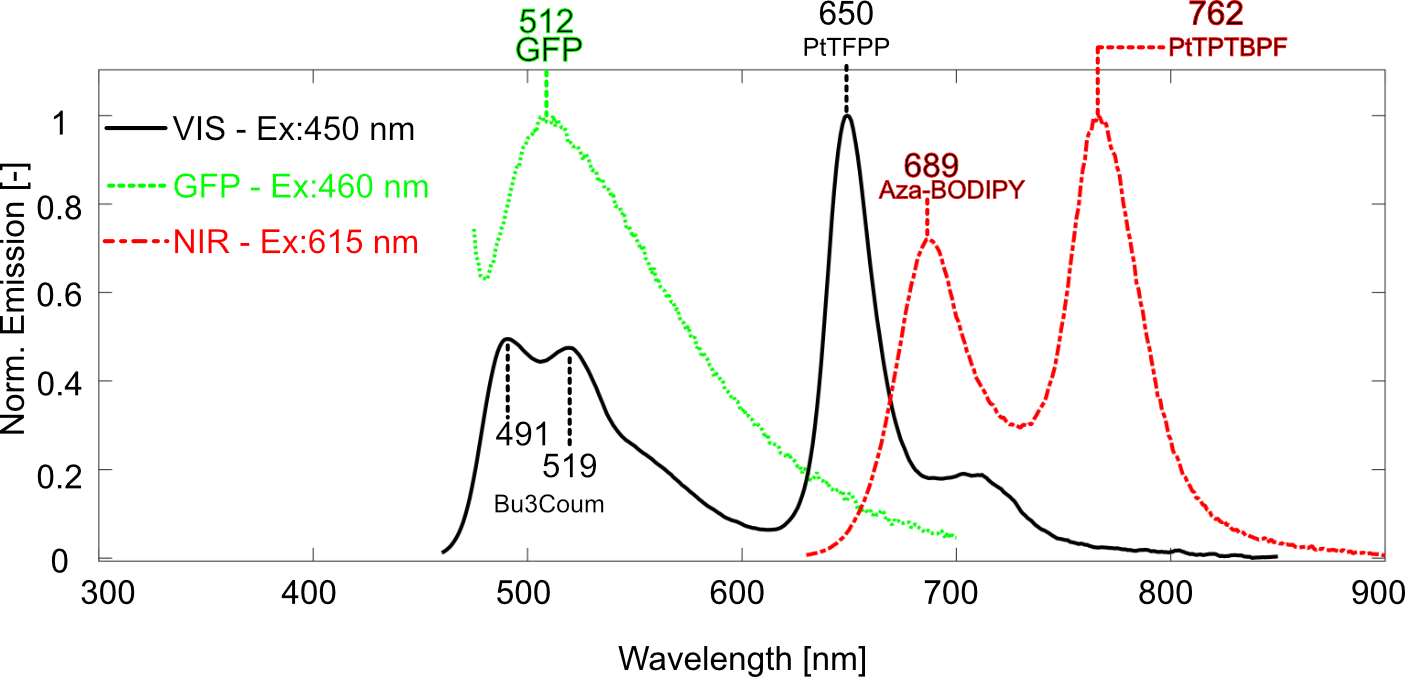


Figure S 2 Comparison of emission spectra of VIS sensor, GFP and NIR sensor. Emission spectra of sensors refer to anoxic conditions. For each element, the excitation wavelength was selected to optimize the emission and is reported in the legend of the figure.

Table S 3 Summary of absorbance peaks of spectra reported in Figure 4 of the manuscript.

| Sample | Dye | Peak wavelength [nm] | Norm. value [-] |
| --- | --- | --- | --- |
| VIS | PtTFPP | 395 | 1 |
|  |  | 508 | 0.07 |
|  |  | 540 | 0.08 |
|  | Bu3Coum | 445 | 0.84 |
|  |  | 464 | 0.73 |
| NIR | PtTPTBPF | 432 | 0.57 |
|  |  | 618 | 0.70 |
|  | Aza-BODIPY | 658 | 1 |

Table S 4 Summary of the emission peaks reported in Figures 4 and 5 of the manuscript under anoxic conditions.

| Sample | Excitation [nm] | Peak wavelength [nm] | Norm. value |
| --- | --- | --- | --- |
| VIS (Figure 4C) | 450 | 491 | 0.49 |
|  |  | 519 | 0.48 |
|  |  | 650 | 1 |
| VIS + mScarlet-I (Figure 5A-B) | 540 | 607 | 1 |
|  |  | 648 | 0.71 |
|  | 450 | 495 | 0.64 |
|  |  | 519 | 0.76 |
|  |  | 559 | 0.51 |
|  |  | 650 | 1 |
| NIR (Figure 4F) | 615 | 689 | 0.72 |
|  |  | 762 | 1 |
| NIR + mScarlet-I (Figure 5C-D) | 540 | 592 | 0.48 |
|  |  | 694 | 1 |
|  | 615 | 692 | 0.72 |
|  |  | 763 | 1 |

# Full maps and image processing details

**Fluorescent strain maps.** The *P. putida* and *P. veronii* maps (Figure 6B–M) are raw 14-bit black-and-white images exported from Axiovision 4.8, with LUT rescaled to each image’s min–max values and displayed using green (GFP) or red (mScarlet-I) colormaps. Background noise was removed with Fiji ImageJ’s color thresholding.

**Oxygen maps** (Figure 6O–Q) were generated as the ratio of O₂-sensitive to reference dye signals and converted to concentrations via the calibration curve (Section S2). Camera noise was removed with MATLAB’s *medfilt2* (5×5).

**Solid grains.** A binary mask, derived from thresholded images taken in bright-field to identify grain perimeters and hole filling in Fiji ImageJ, set pixels of the porous medium geometry to –1.

To show microscale strain distribution and O₂ dynamics, Figure 6 presents zoom-ins of one location at three timesteps. Here, we provide the full maps of *P. putida*, *P. veronii*, and O₂ for all timesteps, showing biomass and O₂ distribution along the flow direction. To reconstruct the full maps (Figure S 3), 80 images per timestep were stitched together. For the GPF and mScarlet-I signals, raw 14-bit grayscale maps are presented with a green or a red color scale, respectively. Oxygen maps are processed identically to the zoom-ins in Figure 6.

**Biomass and chemical imaging resolution.** The spatial resolution of biomass maps is 1 μm per pixel, corresponding to the optical resolution of the imaging system as determined by the camera pixel size and the objective magnification. This value represents the upper bound of the achievable spatial resolution for chemical imaging.

The effective spatial resolution of O_2_ measurements is governed not only by the optical system but also by physico-chemical and optical processes within the sensing layer, as well as by image post-processing. In particular, lateral diffusion of O_2_ within the sensing film, vertical exchange at the sensor–fluid interface, and local biological consumption collectively introduce spatial averaging of the signal. Lateral diffusion is only one of several factors affecting chemical imaging resolution. Additional contributions arise from light guiding within the sensing layer, camera noise, and image post-processing, all of which are known limitations of luminescent optodes [2]. As a result, the chemical imaging resolution is inherently lower than the optical resolution and cannot be defined solely by pixel size. Experimental studies investigating cross-talk in planar optodes suggest that, under worst-case conditions corresponding to sharp step-like oxygen gradients, the effective spatial resolution may approach approximately five times the sensing layer thickness [3]. In our case, this would correspond to ~25 μm. However, such abrupt gradients are unlikely in biologically generated oxygen fields, which are typically smoother and diffusion-regulated. Considering the sensing layer thickness together with the 5 μm Gaussian filtering applied during image processing, we therefore estimate a practical spatial resolution on the order of ~10–15 μm for the present system.


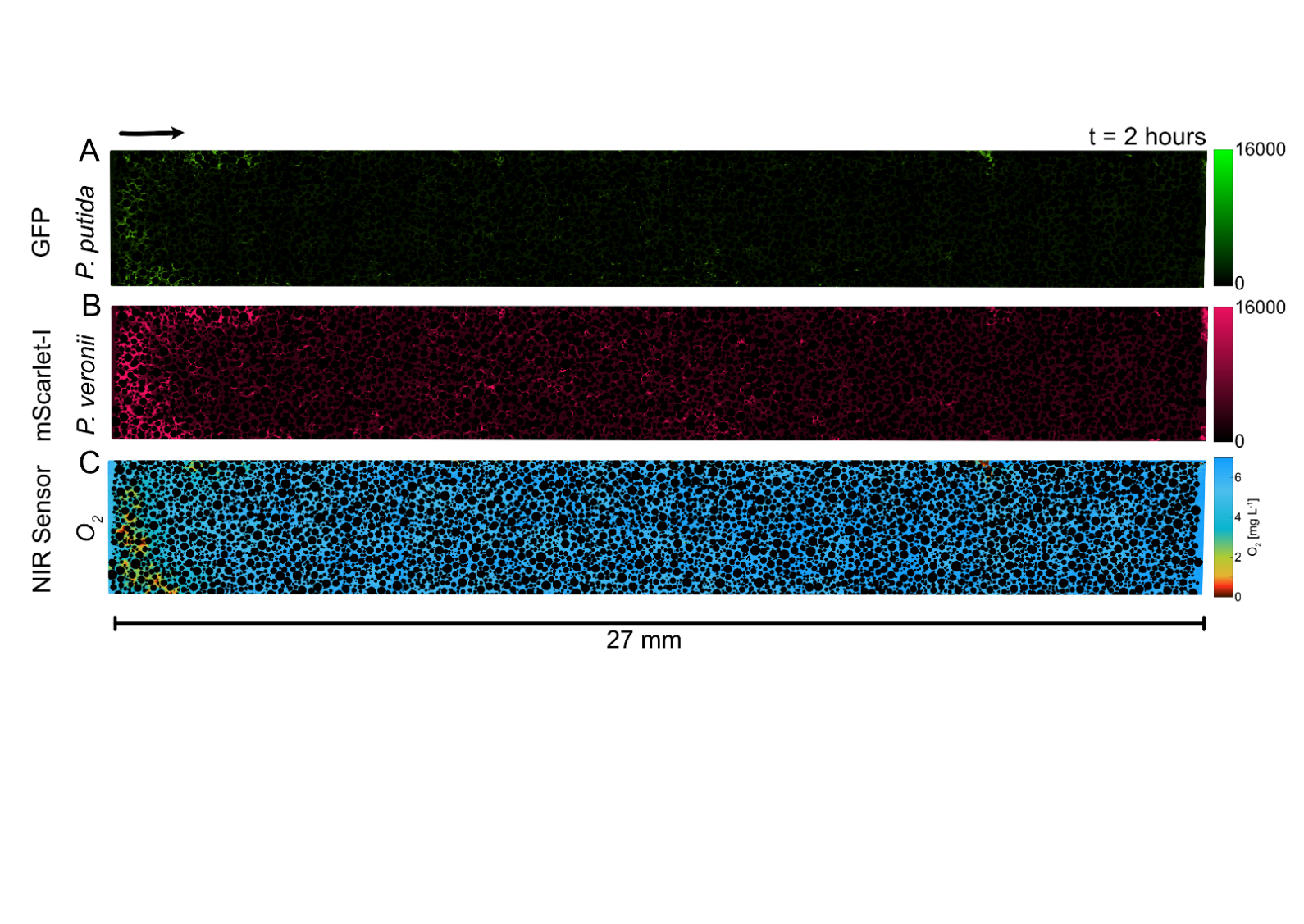


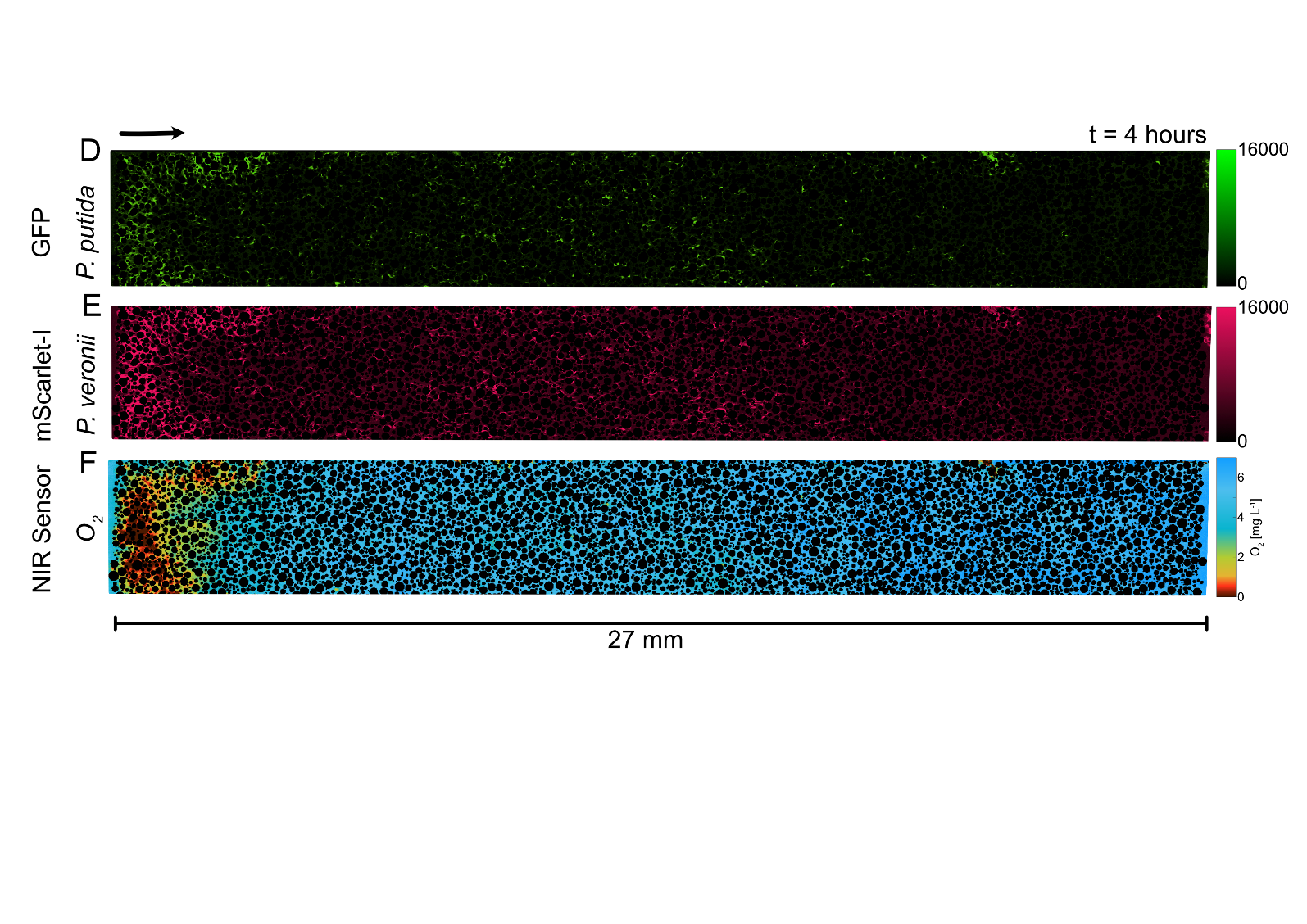

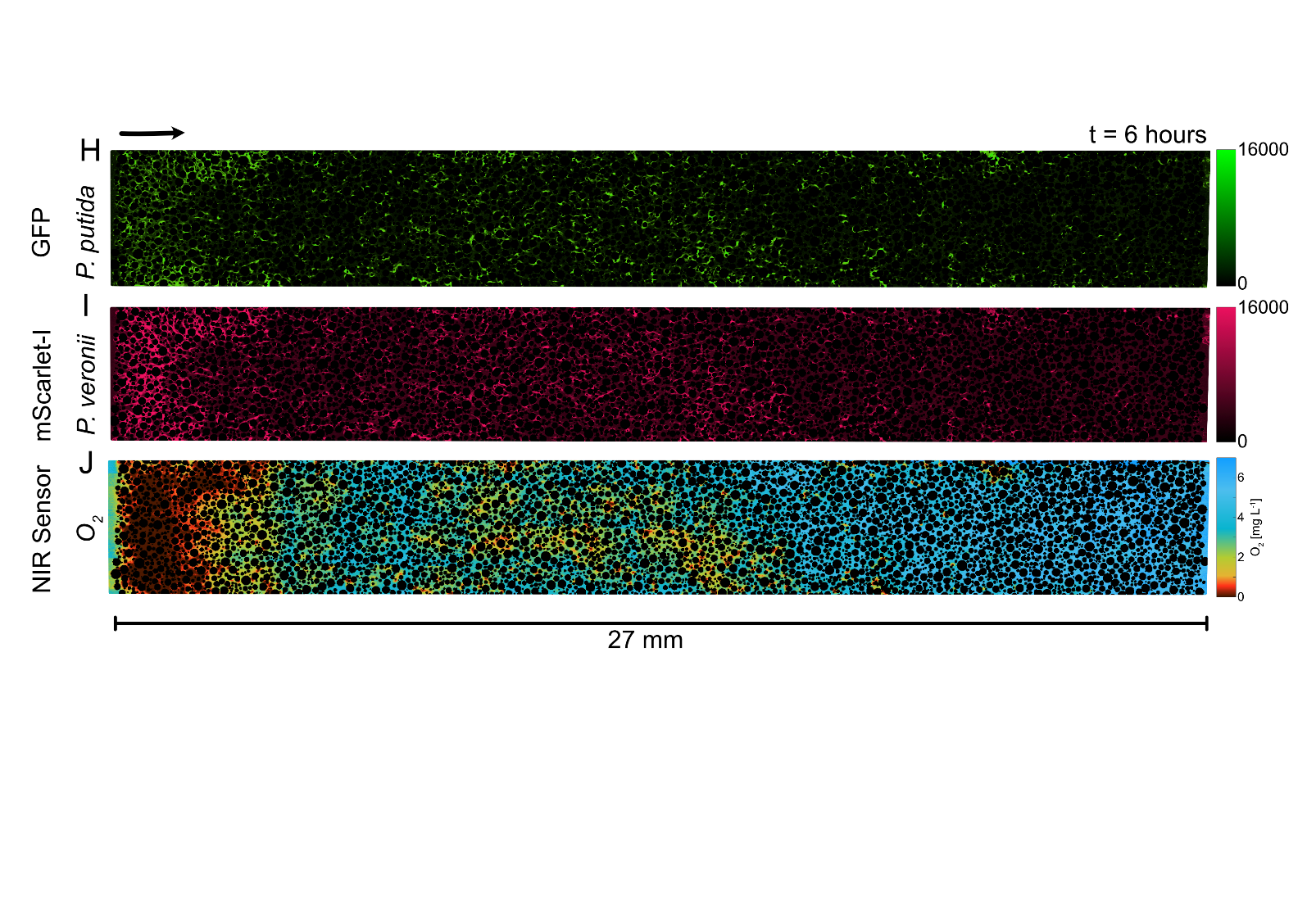

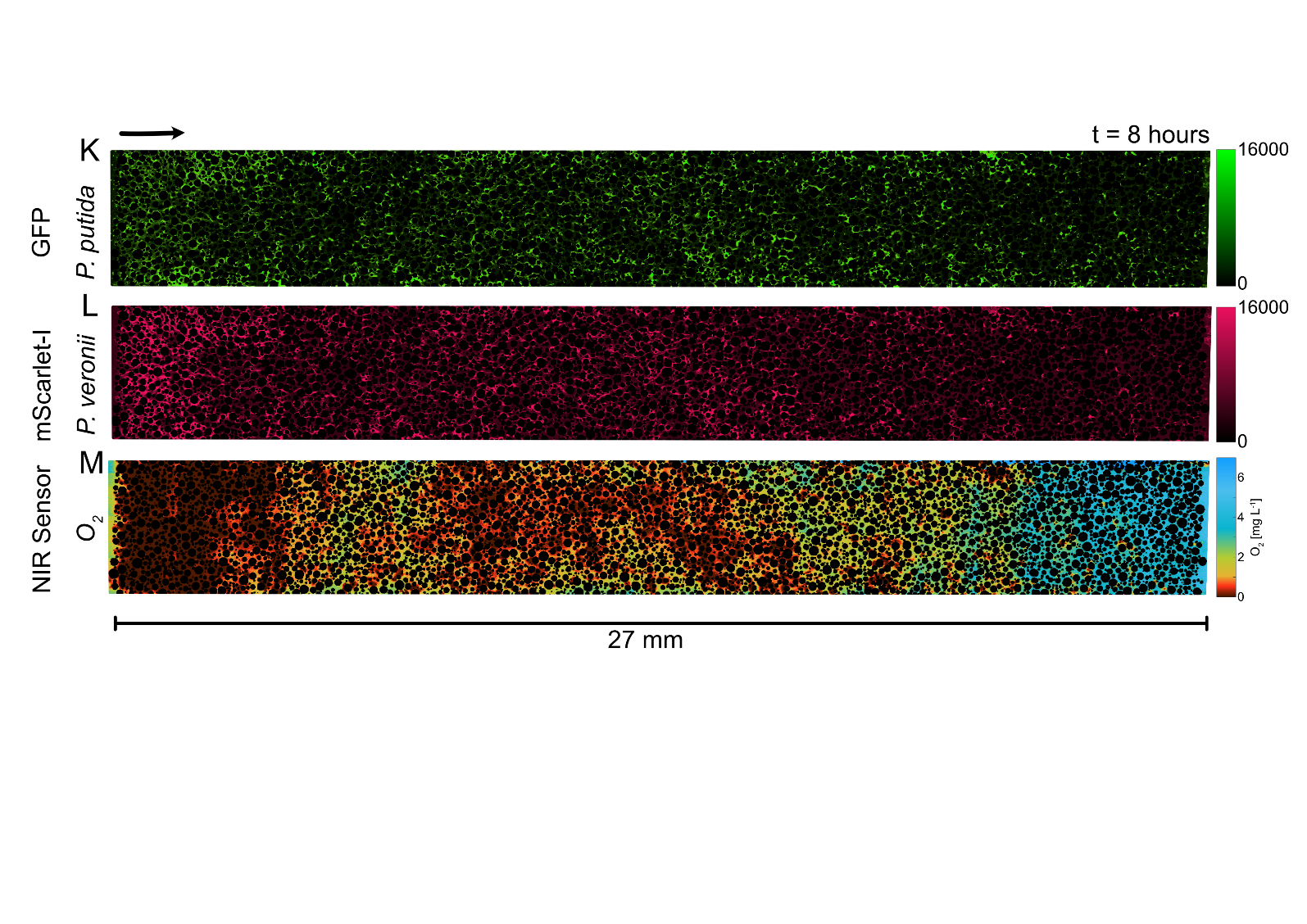

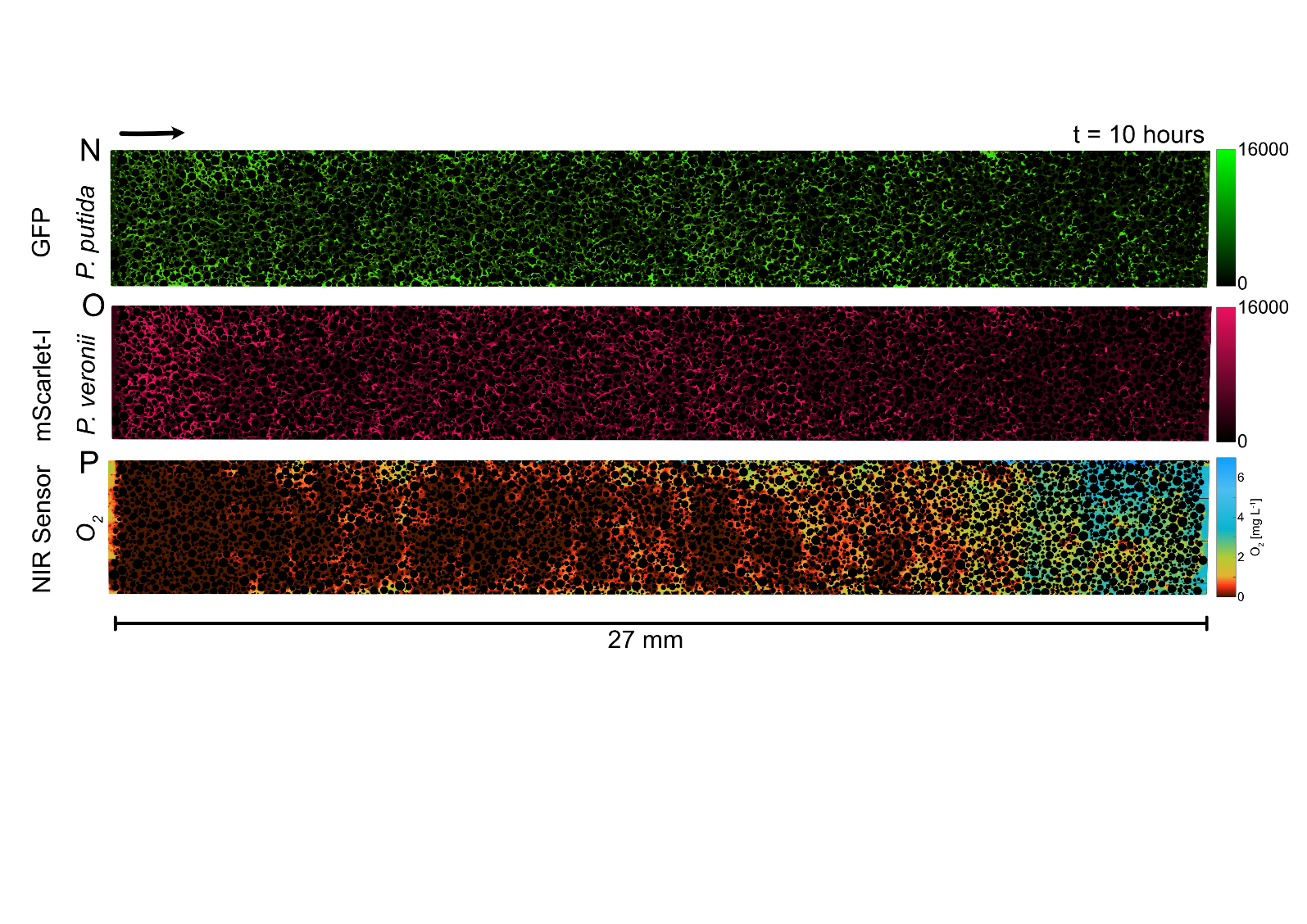

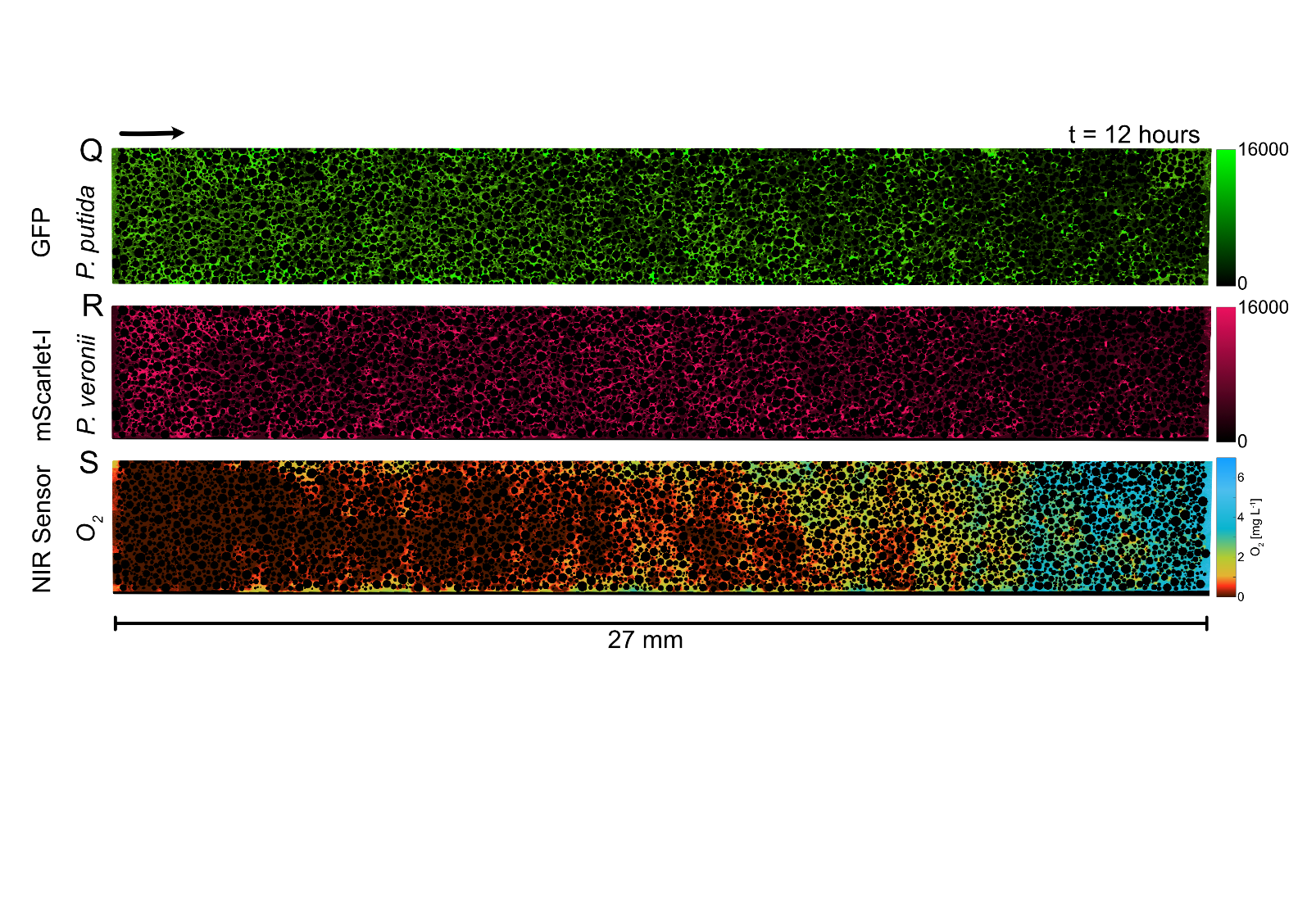


Figure S 3 Maps of GFP P. putida, mScarlet-I P. veronii and O_2_ concentration at t = 2 h (**A ,B , C**), t = 4 h (**D, E, F**), t = 6 h (**G, H, J**), t = 8h (**K, L, M**), t = 10 h (**N, O, P**), and t = 12 h (Q, R, S) after the onset of the flow.

It is worth noting that O_2_ depletion was observed primarily near the inlet, closely following the progression of biomass colonization along the flow direction. In contrast, toward the outlet, the pore water returned to O_2_-rich conditions. This pattern can be explained by the structure of the microfluidic device, which is made of PDMS, a material permeable to gases. As a result, a large portion of the device is exposed to ambient air, allowing O_2_ to diffuse through the PDMS into the microchannel. Previous studies have quantified the diffusivity of O_2_ in PDMS, reporting values comparable to those in pure water (D ≈ 3.2 × 10⁻⁹ m² s⁻¹ [2]), which corresponds to a characteristic diffusion timescale of approximately 10–15 minutes across a ~5 mm PDMS layer. Consequently, only regions of dense bacterial colonization can outcompete the O_2_ flux from the PDMS walls. Areas of the pore space that were sparsely colonized were rapidly re-oxygenated, even when located downstream of anoxic zones.

# Pearson correlation coefficient

The Pearson correlation coefficient is a standard metric to assess the spatial correlation of two variables in space. It varies between -1 and 1. The closer the value is to one, the more positively correlated are the two variables of interest. In other words, high values of the variables are often co-occurring in the same spatial location. We applied the definition of the Pearson correlation coefficient (PCC), assessed pixel by pixel using intensity of GFP (referred to as Green) and mScarlet-I (referred to as Red) signals as entry variables:

$$PCC \left( t \right)= \frac{\sum_{i=1}^{N} \left( \left( {Green}_{i}-E\left[ Green \right] \right)\left( {Red}_{i}-E[Red] \right) \right)}{\sqrt{\sum_{i=1}^{N} \left( {Green}_{i}-E[Green] \right)^{2}\sum_{i=1}^{N} \left( {Red}_{i}-E[Red] \right)^{2}}}$$

where *i* is the pixel identifier, *N* is the total number of pixels of the maps (N = 1.1 x 10^8^), and E[ξ] indicates the mean of the signal ξ (Red or Green) over the entire space. Note that pixels falling in the locations of solid grains, identified by the mask matrix (see Section S4) were excluded from the computation.

At each time step, the PCC value varies as the biomass distribution of both *P. veronii* and *P. putida* changes (Table S 5).

Table S 5 The values of the PCC calculated at each time step of observations.

| Time, t [h] | 2 | 4 | 6 | 8 | 10 | 12 |
| --- | --- | --- | --- | --- | --- | --- |
| PCC [-] | 0.63 | 0.68 | 0.62 | 0.55 | 0.49 | 0.51 |

# Background, biomass threshold, and high-density biomass thresholds

**Background values** were defined as the signal detected in the absence of biomass. To identify these values, we calculated the *probability density function* (*pdf*) of the Green and Red signal in the pore space at t = 2h (Figure S 4). At this time, the background value characterized the largest number of pixels. Therefore, we assigned as the background values those corresponding to the peak of the *pdf*, i.e., 845 and 1390 for Green and Red signals, respectively.

**Biomass threshold and percentage of pore space colonized.** It is reasonable to assume that the background signal is distributed symmetrically around its peak value, which would represent the mean of a Gaussian distribution. Consequently, we assumed that the fluorescent signal was indicative of the presence of biomass only for values outside of the background Gaussian distribution. Exploiting the symmetry of the Gaussian distribution, we could use the half part lower than the mean to size the full interval covered by the Gaussian distribution. As a result, green and red signals were considered associated with the presence of biomass for intensities above 1360 and 2180, respectively (see Figure S 4). These thresholds were referred to as Green_BG_ and Red_BG_ in the main manuscript.

A similar approach was employed to define the background and the biomass thresholding value for phase contrast images, which led to the following values: 1136 as background value and 2190 as biomass threshold.

These values were used to threshold the Green signal, Red signal, and phase-contrast images to quantify the percentage of pore space colonized by *P. putida* and *P. veronii* reported in Figure 7A in the main manuscript.

**High-density biomass threshold.** We defined as high-density biomass regions those that have a value of fluorescent signal higher than Green_HD_ = 4080 and Red_HD_ = 6540, which corresponds to three times the background threshold, defining the presence of biomass.


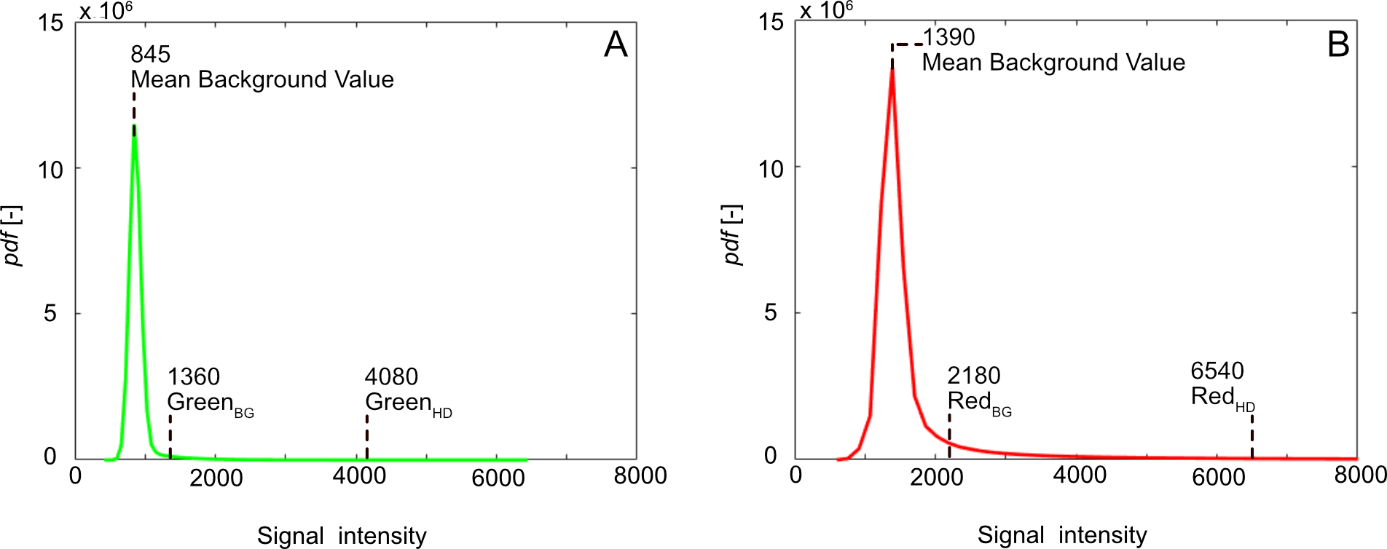


Figure S 4 Probability density function (pdf) of Green (A) and Red (B) fluorescent signal at t = 2h, and the thresholds used in the study to identify the mean background values, the presence of biomass (Green_BG_ and Red_BG_), and the presence of high-density clusters (Green_HD_ and Red_HD_). The signal intensity is expressed as pixel saturation for a 14-bit black and white image. The X-axis, which has an actual length of >16000, is cut to 8000 for illustrative purposes.

# Conditional *probability density functions (pdfs)*


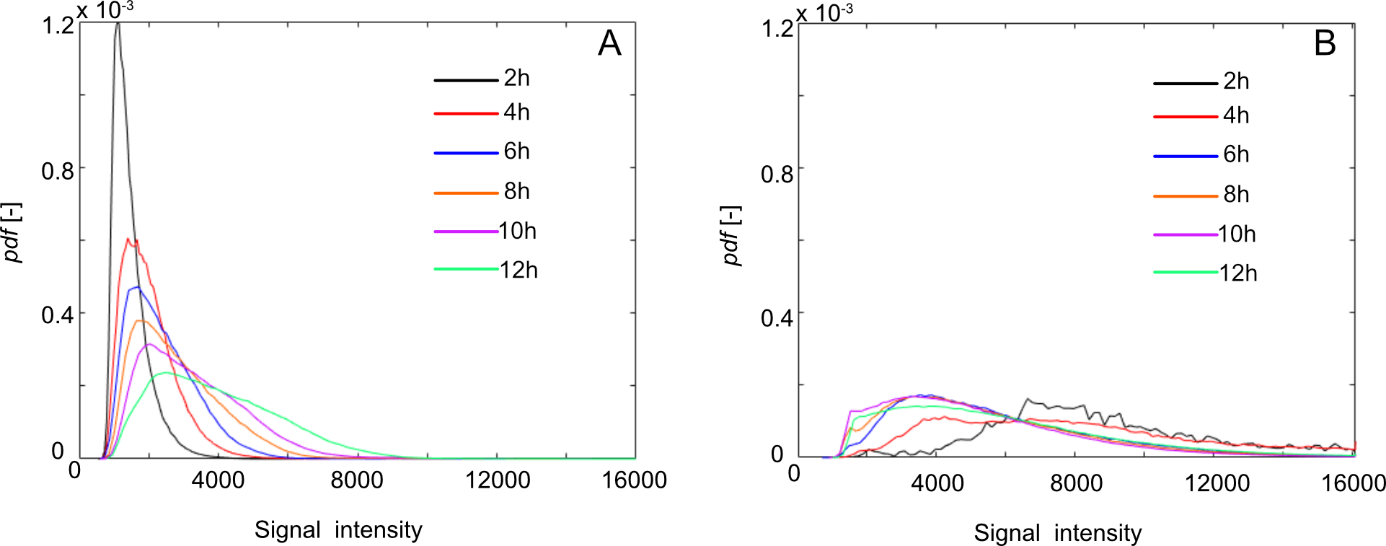


Figure S 5Probability density functions (pdf): A) Green signal pdf conditional to high density of P. veronii; B) Red signal pdf conditional to high density of P. putida at different time steps.

The behavior of conditional *pdf*s has been explored by testing Green_HD_ and Red_HD_ at twice or four times the background threshold. Varying this threshold didn’t affect the relative trend observed for the mean of the conditional *pdf*s presented in Figure 7C in the main manuscript.

# Circularity

Morphological analysis was performed on high-density biomass clusters. This choice assumed that the impact of cell traits on cluster morphology arises only from the collective action of many cells. At each time step, all clusters of pixels with a signal intensity higher than Red_HD_ and Green_HD_ were identified in *P. veronii* and *P. putida* maps, independently, using the MATLAB *bwconncomp* function. We calculated the circularity parameter using the MATLAB *regionprops* function. Circularity can vary between 0 and 1: the higher the value, the more rounded is the morphology (Table S 6).

Table S 6 Values of circularity computed for P. veronii and P. putida aggregates as a function of time.

| Time, t [h] | 2 | 4 | 6 | 8 | 10 | 12 |
| --- | --- | --- | --- | --- | --- | --- |
| Circularity P putida clusters[-] | 0.25 | 0.19 | 0.23 | 0.47 | 0.41 | 0.32 |
| Circularity P veronii clusters[-] | 0.21 | 0.22 | 0.23 | 0.23 | 0.24 | 0.24 |

# Single-cell visualization under changing O_2_ concentrations

The fluorescent signal of bioengineered bacteria is often weak and sensitive to bleaching. Normally, bacterial clusters are more easily detectable as they combine the fluorescent signal of many cells. In some microbial ecology studies, however, it is essential to observe the behavior of single cells. Therefore, we verified that the presence of the sensor was not preventing the visualization of isolated fluorescent cells suspended in a mixed community, using application 2, described in the main text. We report here the images of the straight channel along the chemically generated O_2_ gradient, demonstrating the visibility of single fluorescent cells despite the presence of the O_2_ sensor and its increasing signal intensity with decreasing O_2_ concentrations.

Oxygen maps were processed as done for application 1, described in Section S4. To identify bacterial cells, adaptive thresholding has been applied using the *adaptthresh* function of MATLAB with a sensitivity parameter equal to 0.3 and 0.4 for GFP and mScarlet-I signals, respectively.


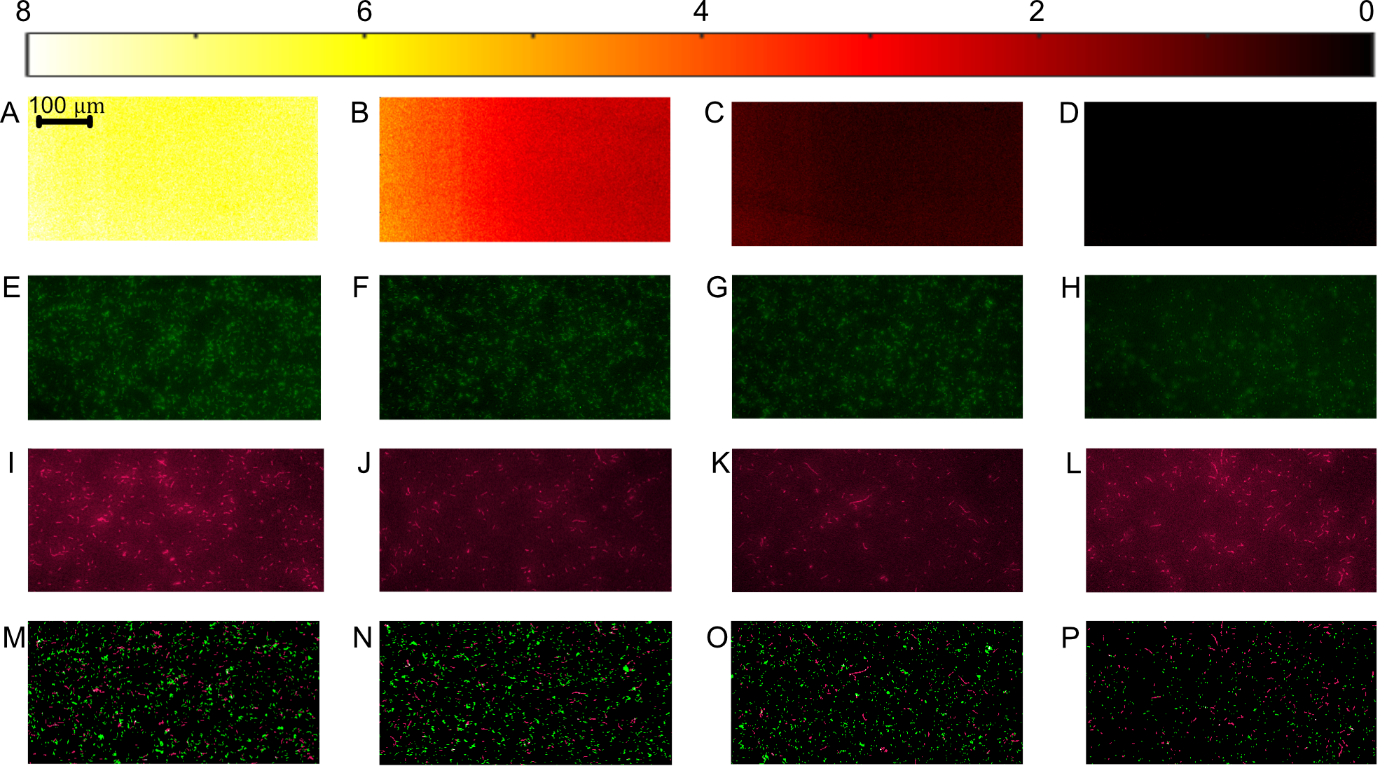


Figure S 6 Maps of O_2_ concentration in different locations along the chemically generated O_2_ gradient (A-D) and the corresponding raw images of GFP signal (E-H), raw images of mScarlet-I signal (I-L), and the resulting distribution of P. putida and P. veronii (M-P). The raw images are originally in 14-bit greyscale and are reported here in green and red color scales for the GFP and mScarlet-I signals, respectively.

# Growth rates in batches

*P. putida* and *P. veronii* were cultivated in a batch incubation at 23 °C (same laboratory temperature as the microfluidic experiment) in Erlenmeyer flasks under well-mixed oxic conditions maintained by a magnetic stirrer. A cotton lid isolated the cultures from external contamination. The cultivation medium contained 50% v/v Luria-Bertani broth, i.e., the same medium used in the microfluidic flow experiment. The initial inoculum was prepared by diluting 50 times the overnight cultures, grown from frozen stocks at 30 °C in an orbital shaker at 180 rpm. An aliquot of 300 μL was sampled from the batch incubation at regular intervals using a pipette under a laminar flow hood to avoid contamination during sampling. Bacterial growth was measured via optical density (OD_600_, Figure S 7) using a TECAN Spark spectrophotometer (96-well plates). All material used in the experiment was sterilized via autoclaving at 120 °C for 20 minutes.

Experimental data were fitted to a logistic curve using the *cftool* app in MATLAB (Table S 7).


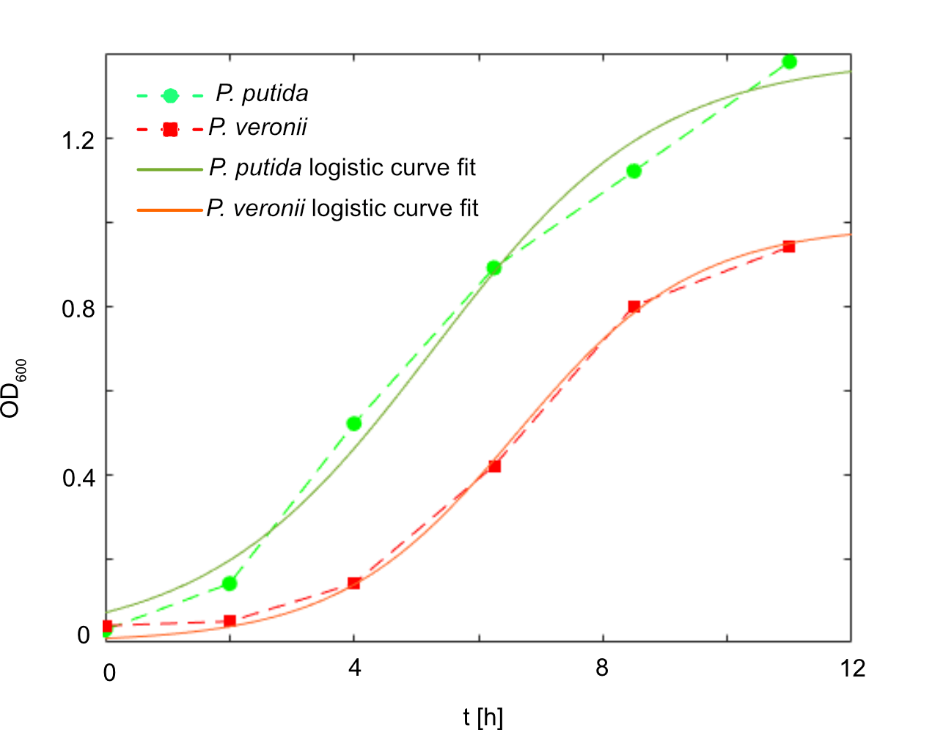


Figure S 7 Optical densities measured at regular time intervals in oxic well-stirred batch incubations for P. putida and P. veronii. Continuous lines indicate the fitted logistic curves.

Table S 7 Parameters of the logistic curve estimated for P. putida and P. veronii.

| Strain | L (carrying capacity) | K (logistic growth rate) | X_0_ (function midpoint) |
| --- | --- | --- | --- |
| *P. putida* | 1.4 | 0.6 | 5.3 |
| *P. veronii* | 1.0 | 0.7 | 6.6 |

# A combination of 4 fluorescent proteins compatible with the NIR sensor

In this study, we tested the compatibility of mScarlet-I and GFP fluorescent proteins with the NIR O_2_ sensor. However, bioengineering has already developed hundreds of different fluorescent proteins, with variable emission wavelengths distributed across the visible part of the spectrum. Here we propose a combination of 4 different fluorescent proteins that present distinct emission peaks, without interfering with the NIR O_2_ sensor emission (Figure S 8). All the fluorescent proteins proposed here, namely mTagBFP2 [3], GFP (this study), mBanana [4], mScarlet-I (this study), have already successfully been bio-engineered and expressed in bacterial cells. Therefore, they are likely employable in microbial ecology studies along with O_2_ sensors.


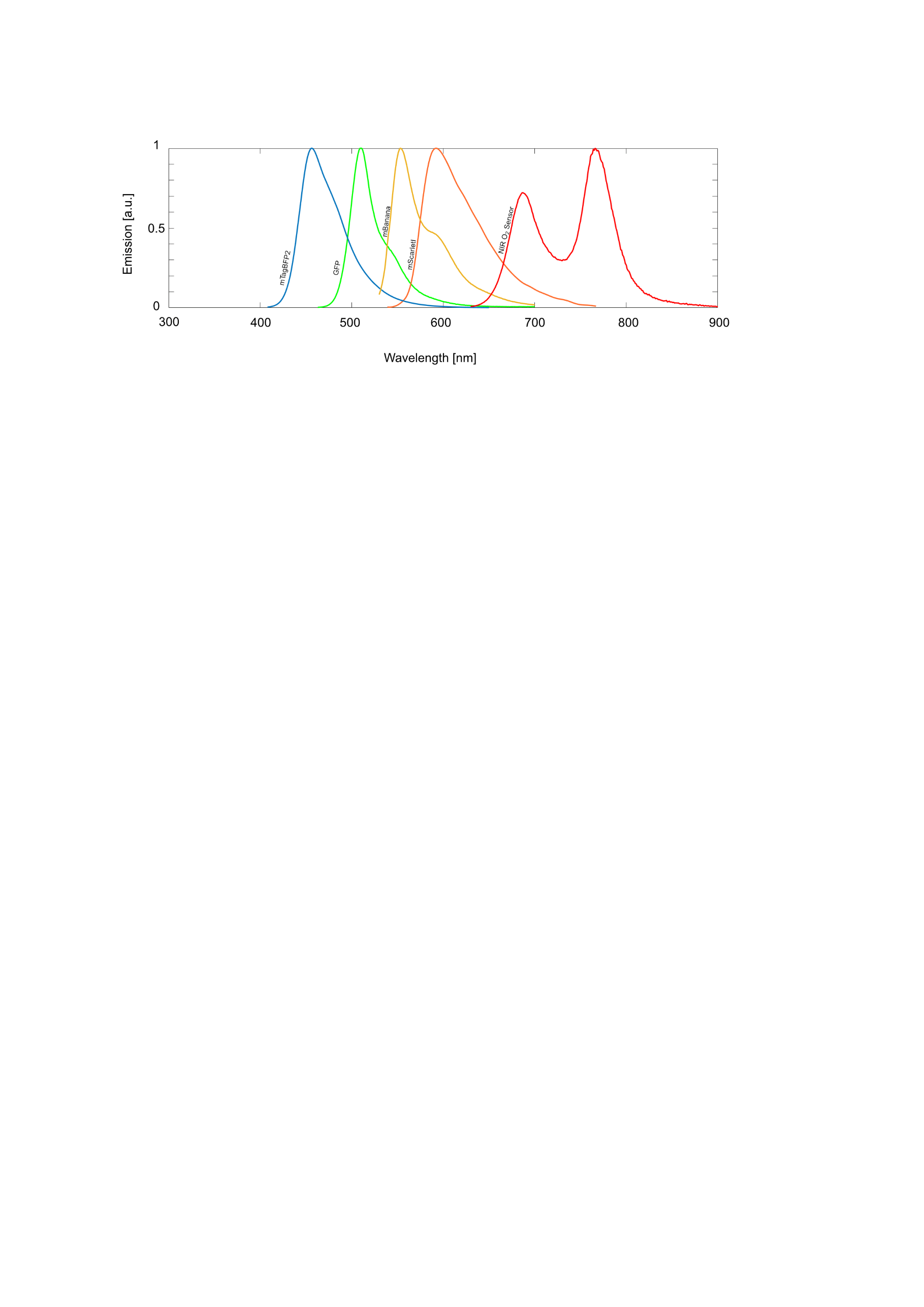


Figure S 8 Emission spectra of four different fluorescent proteins available for cell bioengineering, taken from [https://www.fpbase.org/,](https://www.fpbase.org/) along with the emission spectrum of the NIR O_2_ sensor used in this study.

1. Morrison, G., C. KL, and M. Grasserbauer, *GENERAL ASPECTS OF TRACE ANALYTICAL METHODS. IV: RECOMMANDATIONS FOR NOMENCLATURE, STANDARD PROCEDURES AND REPORTING OF EXPERIMENTAL DATA FOR SURFACE ANALYSIS TECHNIQUES.* 1979.

2. Markov, D.A., et al., *Variation in diffusion of gases through PDMS due to plasma surface treatment and storage conditions.* Biomedical microdevices, 2014. **16**: p. 91-96.

3. Seo, P.-W., G.-J. Kim, and J.-S. Kim, *A short guide on blue fluorescent proteins: limits and perspectives.* Applied Microbiology and Biotechnology, 2024. **108**(1): p. 208.

4. Ding, Y., et al., *Crystallization and Preliminary X-ray Analysis of Fluorescent Protein mBanana.* Protein and Peptide Letters, 2008. **15**(1): p. 113-114.
